# Integrated Operational Taxonomic Units (IOTUs) in Echolocating Bats: A Bridge between Molecular and Traditional Taxonomy

**DOI:** 10.1371/journal.pone.0040122

**Published:** 2012-06-28

**Authors:** Andrea Galimberti, Martina Spada, Danilo Russo, Mauro Mucedda, Paolo Agnelli, Angelica Crottini, Emanuele Ferri, Adriano Martinoli, Maurizio Casiraghi

**Affiliations:** 1 ZooPlantLab, Dipartimento di Biotecnologie e Bioscienze, Università degli Studi di Milano-Bicocca, Milano, Italy; 2 Dipartimento Ambiente-Salute-Sicurezza, Università degli Studi dell’Insubria, Varese, Italy; 3 Laboratorio di Ecologia Applicata, Dipartimento Ar.Bo.Pa.Ve., Facoltà di Agraria, Università degli Studi di Napoli Federico II, Portici (Napoli), Italy; 4 Gruppo Speleologico Sassarese e Centro Pipistrelli Sardegna, Sassari, Italy; 5 Museo di Storia Naturale dell’Università di Firenze, Sezione di Zoologia “La Specola”, Firenze, Italy; 6 CIBIO, Centro de Investigação em Biodiversidade e Recursos Genéticos, Campus Agrário de Vairão, R. Padre Armando Quintas, Vairão, Portugal; American Museum of Natural History, United States of America

## Abstract

**Background:**

Nowadays, molecular techniques are widespread tools for the identification of biological entities. However, until very few years ago, their application to taxonomy provoked intense debates between traditional and molecular taxonomists. To prevent every kind of disagreement, it is essential to standardize taxonomic definitions. Along these lines, we introduced the concept of Integrated Operational Taxonomic Unit (IOTU). IOTUs come from the concept of Operational Taxonomic Unit (OTU) and paralleled the Molecular Operational Taxonomic Unit (MOTU). The latter is largely used as a standard in many molecular-based works (even if not always explicitly formalized). However, while MOTUs are assigned solely on molecular variation criteria, IOTUs are identified from patterns of molecular variation that are supported by at least one more taxonomic characteristic.

**Methodology/Principal Findings:**

We tested the use of IOTUs on the widest DNA barcoding dataset of Italian echolocating bats species ever assembled (i.e. 31 species, 209 samples). We identified 31 molecular entities, 26 of which corresponded to the morphologically assigned species, two MOTUs and three IOTUs. Interestingly, we found three IOTUs in *Myotis nattereri*, one of which is a newly described lineage found only in central and southern Italy. In addition, we found a level of molecular variability within four vespertilionid species deserving further analyses. According to our scheme two of them (i.e. *M.* *bechsteinii* and *Plecotus auritus*) should be ranked as unconfirmed candidate species (UCS).

**Conclusions/Significance:**

From a systematic point of view, IOTUs are more informative than the general concept of OTUs and the more recent MOTUs. According to information content, IOTUs are closer to species, although it is important to underline that IOTUs are not species. Overall, the use of a more precise panel of taxonomic entities increases the clarity in the systematic field and has the potential to fill the gaps between modern and traditional taxonomy.

## Introduction

Taxonomy is an old discipline that underwent several upgrades in its about 250 years. In the last decades, biological classification schemes have been revised with the inclusion of two relevant innovations: molecularization (i.e. the investigation of variability in molecular markers used as a discriminator) and computerization (i.e. the not redundant transposition of the data using informatics supports) [1]. In modern taxonomy, it remains sometimes controversial whether to include both molecular and morphological characters in the same classification scheme [2]–[5]. Since the advent of molecular-based taxonomy, many studies contributed to define a plethora of new taxonomic entities. In molecular approaches, one of the most relevant entities is the Operational Taxonomic Unit (OTU) [6] that was first defined in a non-molecular context. In its original use, the OTU is defined using as much characters as possible, even without knowing the “real” taxonomic value of each character. In such a context, DNA sequences are the typical data that can be used to define OTUs, because each sequence can be considered as a group of characters, not *a priori* weighted. Afterwards, [7] introduced the concept of Molecular Operational Taxonomic Unit (MOTU) to define those entities identified in a molecular context. In a strict sense MOTU is a subset of an OTU that represents the more comprehensive assemblage. Even if the term MOTU is not completely independent from the concept of OTU, we believe that its introduction is valuable giving promptly information on the origin of the data supporting the entity.

Nowadays, one of the most widely used molecular approaches in species identification is DNA barcoding [8]. Following a strict operational workflow, this technique reveals sequence variation among taxa at short specific genomic regions [8]–[13]. In DNA barcoding literature, the designation MOTU has been widely used to describe “clusters of sequences (that act as representatives of the genomes from which they are derived) generated by an explicit algorithm” [7], [14]. Using a clustering algorithm, MOTUs can be defined by different approaches among which the use of specific cut-off values based on sequences similarity. In DNA barcoding literature MOTU can designate different situations that we here interpreted as belonging to three distinct groupings: (M1) a group of unidentified organisms sharing similar sequences (see for example [7]); (M2) a group of organisms within a species that are distinct at the molecular level from other members of the species (see for example [15]); and (M3) a group of organisms from different species that are similar at the molecular level (see for example [16]).

Here, we propose a synergistic synthesis of classical taxonomic approaches (e.g. morphology, biogeography) and molecular characteristics called Integrated Operational Taxonomic Units (IOTUs). Like MOTUs, IOTUs describe organisms that are similar at the molecular level (i.e. share a DNA barcode), but, unlike MOTUs (considered in its original definition), they also share at least one other characteristic from the ‘taxonomic circle’ [2]. In other words, based on [2] first proposal, we define IOTUs as groups of organisms confirmed by at least two approaches, one of which is molecular-based. It is noteworthy that IOTUs are identified on a molecular base, but the molecular definition is reinforced by diagnostic at other biological characteristics giving to IOTUs higher information content and a stronger taxonomic support.

**Figure 1:**
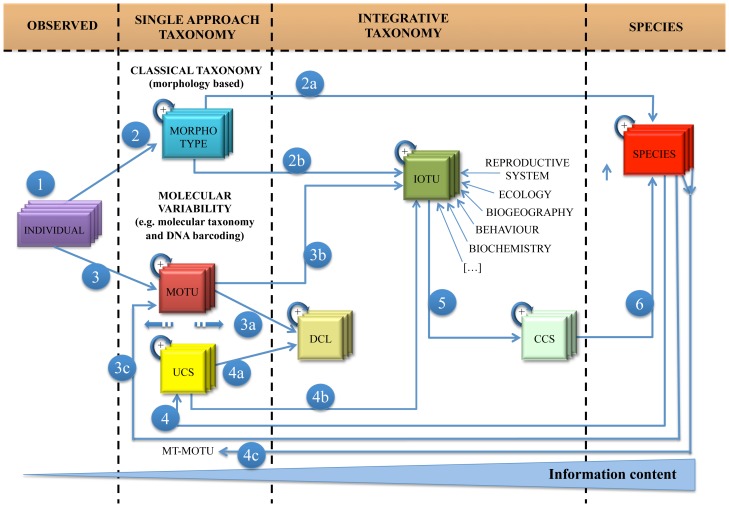
Taxonomic ranks and their relationships in a molecular-based taxonomic study. In this schematic view the taxonomic ranks can be grouped in four different areas discriminated by their information content: individuals lie in the less informative level; a single taxonomic approach identifies morphotypes, MOTU and UCS; integration of data allows the definition of DCL, IOTU and CCS; the last and more informative level contains species. Individuals represent the first level of observation (1). These organisms are grouped on the basis of morphological similarities (2), in a classical taxonomic approach, which may lead to the identification of a species (2a), but can also be one of the inputs of the IOTU (2b). Molecular variability observed among individuals can lead to the definition of MOTUs (3) that, with the addition of more data, can be elevated to the level of DCL (3a) or IOTU (3b). However, in many published works MOTUs are identified within nominal species without additional taxonomic data (3c), being in this sense synonyms of UCS. As a consequence, the information content of MOTU and UCS is variable as identified by the dotted arrows between them. UCS is identified within a species (4), if further taxonomic data are provided it can be elevated to a DCL (4a) or an IOTU (4b). When two or more nominal species are similar at the molecular level for the chosen marker we call this situation Multi Taxa - Molecular Operational Taxonomic Units (MT-MOTUs) (4c). IOTU is the rank reached by a biological entity defined by molecular data coherently coupled with other source of information. When IOTU has reached a sufficient level of information it can be elevated to the rank of a CCS (5), which following a formal description will become species (6). The “+” in the left up corner of each box indicates that within each taxonomic rank, more than a single entity belonging to that rank can occur. MOTU is defined according to [7]; UCS, DCL and CCS are defined according to [17], [18].

This IOTU definition has to be set in the framework of Unconfirmed Candidate Species (UCS), Deep Conspecific Lineage (DCL), and Confirmed Candidate Species (CCS), which are intermediate states between individuals and species that were introduced by [17] and implemented by [18]. According to both studies, UCSs are conspecifics that are separated by “some” (not better definable) genetic distances. In this sense, UCSs are synonymous with MOTU M2 concept and deserve to be further investigated with other approaches in order to clarify their effective taxonomic status. DCLs are UCSs or MOTUs that cannot be further differentiated by additional taxonomic data. By contrast, CCSs have additional taxonomic data confirming that the divergent lineages are true species, but they require a formal description by a taxonomic expert to be accepted and named. Individuals, morphotypes, MOTUs, UCSs, DCLs, IOTUs, CCS, and species are entities that can be ranked in order of increasing information content (Figure 1). The ranking of these entities is the first and essential step to fill the gap between molecular and traditional taxonomists. Indeed, species should be identified and described from as many taxonomic characteristics as possible. Relying on a single approach to define a species can be misleading [19]. For example, the African elephant *Loxodonta africana* was considered a single species mainly on the basis on morphological data but has been reclassified as two species with the inclusion of molecular data [20].

To evaluate this integrated taxonomic approach and to underpin the definition and use of IOTUs, we focused on Italian echolocating bats. As a Mediterranean peninsula, Italy has a high degree of biodiversity [21]. This situation was generated by mountain ranges, such as the Alps and the Apennines, which acted as geographical barriers to dispersal during Pleistocene climatic fluctuations [22]–[27]. As a consequence, a lot of cryptic species have been identified for a wide range of taxa, including bats [22]–[30].

Nowadays, Italy is home to 34 bat species [30]–[33] but, as a general condition for this group of mammals, many taxa are nearly or completely indistinguishable morphologically, acoustically or biometrically [11], [34]–[37]. Although identification keys based on morphological characters and biometric measurements are available for European bats [35], correct use of these keys requires considerable training and experience. With the integration of molecular data into taxonomic studies, the number of molecular lineages within bats has increased significantly at the global scale [19], [31], [38]. In the case of European populations, molecular techniques led to the identification of at least seven new cryptic vespertilionid species [30]–[32].

Vespertilionidae is the most species-rich family in Europe and Italy and is characterized by high levels of cryptic diversity. Four cryptic species have been identified in this family (*Pipistrellus pygmaeus*, *Myotis alcathoe*, *M. punicus*, *and Plecotus macrobullaris*) from Italy or its neighbouring countries [39]–[47]. This family also includes *Plecotus sardus*, which is the only known bat species endemic to Italy [48].

Overall, Italian bat populations have been poorly investigated and require more attention [27], [33]. Moreover, as has been observed across Europe, Italian bat populations have declined and now require monitoring to implement conservation measures [49]. Successful monitoring requires that species be correctly identified to map occurrences and estimate population sizes. With no morphological criteria for identification, cryptic species are lost in these conservation efforts.

To evaluate a new integrated approach for taxonomic schemes, we assembled a reference *coxI* dataset from samples representing 31 of the 34 Italian bat species. Observed variation in *coxI* was then combined with morphological taxonomic assignment made by experienced bat specialists. With this methodological approach we aimed to: *i*) evaluate how the concepts of MOTU and IOTU are related, *ii*) describe the genetic differences between sampled taxa and determine how well these differences correspond to morphological-based taxonomy, *iii*) evaluate how well the integrated approach can be useful in identifying samples belonging to the main cryptic taxa, and *iv*) investigate intraspecific molecular variation of the DNA barcode region to detect divergent lineages within species that are widespread distributed in the Italian peninsula.

## Materials and Methods

### Collection and Identification of Samples

We sampled 209 individuals from 31 bat species at 43 sites across the Italian peninsula and in Sardinia (Table S1). *Nyctalus lasiopterus* and *Vespertilio murinus* were excluded due to their rarity in the peninsula [33]. To maximize the chance to observe intraspecific geographic variation, conspecifics were sampled from distant sites. All experiments, procedures and ethical issues were conformed to the competent national ethical bodies: most samples were obtained from field-caught bats in mist-nets or bat-boxes under license from the Italian authorities (Protocol n. 004612/T-A 31 of February 3, 2009 released by the Institute for Environmental Protection and Research, and approved by the Italian Ministry for Environment, Territory and Sea). The remaining samples were specimens in the collection of the Natural History Museum of Florence University (Zoological Section “La Specola”; MZUF). In most cases, individual bats examined in the field were mostly recognized to species level by taking standard linear measurements with a 0.1 mm precision digital caliper and/or assessing the species-specific occurrence of diagnostic criteria following the most updated identification keys [35]. The application of such standard criteria is widespread among bat researchers in Europe and validated both in Italy and in the rest of the Europe: they are thus well known to confidently separate most European species.

Forty-one samples came from cryptic species of *Myotis*, *Plecotus*, and *Pipistrellus*, and overlapping morphological characters between congeners made specific identification impossible. As far as the *Myotis mystacinus* group was concerned, although *M. brandtii* was diagnosed based on tooth morphology and penis shape [35], we made no *a priori* distinction between *Myotis mystacinus* and *Myotis alcathoe* whose morphological discrimination may not be obvious. For cryptic *Pipistrellus pipistrellus/P. pygmaeus*, to obtain a further piece of diagnostic information we also recorded echolocation calls on release with a Pettersson D1000X detector in the direct ultrasound sampling mode (sampling rate was 500000 Hz). At least three echolocation calls/sequences were analyzed with BatSound rel. 4: we generated spectrograms and power spectra (1024 pt. FFT size, 98% window overlap) to take end frequency and frequency of maximum energy to help separate such species as done in previous studies regarding the Italian territory [36], [43].

Using a biopsy punch, a 3-mm diameter sample was taken from each wing membrane for genetic analysis. For museum samples, 50 mg of muscular tissue was stored in 99% ethanol. Following the protocol specified by the Biorepositories Initiative (http://www.biorepositories.org), all samples were catalogued as MIB:zpl.

### DNA Extraction, PCR Conditions, DNA Sequencing and Alignment

We extracted total genomic DNA from a single ‘punch’ or 25 mg of muscular tissue using guanidinium thiocyanate and diatomaceous earth [50]. To amplify the 658 bp target region of *coxI*, we used primers VF1d (5′-TTCTCAACCAACCACAAR GAYATYGG-3′) and VR1d (5′-TAGACTTCTGGGTGGCCRAARAAYCA-3′) [51] in a 20-µl PCR containing 1X MasterTaq buffer with 1.5 mM MgCl_2_ (Eppendorf AG, Hamburg, Germany), 0.2 mM of each dNTP, 1 µM of each primer, 1 U of MasterTaq DNA polymerase (Eppendorf AG, Hamburg, Germany) and 1–10 ng of template DNA. The PCR used the following cycling conditions: 1 min at 94°C, followed by 5 cycles of 30 s at 94°C, 40 s at 50°C, and 1 min at 72°C, followed by 35 cycles of 30 s at 94°C, 40 s at 55°C, and 1 min at 72°C, and ending with 10 min at 72°C [11]. PCR products were gel-purified using the Perfectprep Gel Cleanup (Eppendorf AG, Hamburg, Germany) and sequenced directly on an ABI3730XL automated sequencer (Macrogen Inc., Korea) with both PCR amplification primers.

Sequences were checked by eye and edited manually with BioEdit sequence alignment editor (version 7.0.5 [52]), and trimmed to yield the same length for all entries in the final alignment.

To avoid the inclusion of *coxI* nuclear pseudogenes of mitochondrial origin (i.e. NUMTs [53]), we followed the guidelines proposed in [54] and [55]. Sequences were deposited into the EMBL Data Library under the accession numbers [EMBL: FR856638 - FR856846] (Table S1).

A different treatment was adopted for the investigation of *M.* *nattereri* samples. Although easily differentiated from its congenerics [35], recent genetic studies conducted on Western Palearctic populations have reported the occurrence of at least four deeply divergent intraspecific lineages for *M. nattereri* [30], [56], [57]. Except for *M. escalerai* [58] that has been recently split as a different species, the other lineages (one of which is distributed in northern Italy) lack of a detailed taxonomic assessment. Moreover, any molecular study conducted on this taxon was performed with a standardized DNA barcoding approach and very few data are available for *M.* *nattereri* populations of southern Italy. Given these assumptions, we decided to compare sequences from morphologically recognized *M. nattereri* of northern and southern Italian populations with closely related lineages, including *M. escalerai*, from other sites in the Western Palearctic (Table S2). To do this, due to the scarce presence in GenBank of *coxI* data for the *M. nattereri* complex, we amplified portions of mitochondrial ND1 and *cyt b* using published PCR conditions [30]. Sequences were deposited into the EMBL Data Library under the accession numbers [EMBL: FR856847 - FR856854]. These *loci* had been previously sequenced in *M. nattereri* and related taxa from outside of Italy.

### DNA Barcoding Datasets, Optimum Threshold (OT) Calculation, and Designation of Taxonomic Ranks

To evaluate how well DNA barcoding distinguishes named Italian bat species, we measured the correlation between morphologically identified species and the *coxI* genetic divergence for each species. Pairwise evolutionary distances were calculated by the Kimura 2-Parameter (K2P) method [59] in MEGA 4.0 [60]. The extent of genetic differentiation between and within species was calculated by averaging pairwise comparisons of sequence divergence across samples as described in [38].

We generated two datasets with the *coxI* sequences: the reference dataset and the comprehensive dataset. The former was an alignment of *coxI* sequences from individuals that were classified to the species level in the field and 14 *coxI* sequences from seven European vespertilionid species from GenBank marked as ‘barcode standard’ (Table S1; see also [61]). We excluded *M.* *nattereri* because of taxonomic uncertainties regarding this taxon [30], [56].

The second dataset (i.e. comprehensive dataset) encompassed all the *coxI* sequences amplified for this study, including those in the reference dataset, the 22 *M. nattereri* sequences, and 41 sequences from morphologically unidentified specimens from *Myotis*, *Plecotus* and *Pipistrellus*.

With the reference dataset, we evaluated how well the morphological identification and molecular variations were correlated. Using this dataset, we calculated the Optimum Threshold (OT) using a PERL script developed by [16]. OT is a value of molecular divergence, directly deriving from the whole range of molecular variability in the reference dataset. This threshold value maximizes the coherence between the morphological-based identification and the molecular variability in the barcode region minimizing, at the same time, the total amount of identification mismatches that could occur when data obtained with the two approaches are compared. Identification mismatches could include Type I errors (i.e. when molecular variability values higher than OT are found among conspecifics) and Type II errors (i.e. when different species, show values of molecular variability lower than OT). The lower is OT, the higher is the probability to deal with Type I errors, while high values of OT generally correspond to a high percentage of Type II errors. The sum of both error contributions represents the so-called “cumulative error” (CE), and when the minimum cumulative error value (MCE) is reached the OT is found.

Using a DNA barcoding approach, we categorized the Italian bat species into the ranks depicted in Figure 1 (e.g. MOTUs, IOTUs, UCS).

### Identification of Unclassified Samples and Detection of Cryptic Lineages

We analyzed the K2P distance matrix from the comprehensive dataset to perform two different analyses. First, we classified the 41 morphologically unidentified samples into species groups (i.e. DNA barcoding) by comparing their barcode sequences with those included in the reference dataset. Then, we compared these identification results with those obtained using the Identification Engine tool (IDS) in the Barcode of Life Data System (BOLD: http://www.barcodinglife.org/; Species Level Barcode Records database), which returns unique species assignments based on ≥99% sequence similarity at the barcode sequence [62]. Second, we used the K2P distance matrix to reveal geographic lineages or new cryptic taxa (i.e. DNA taxonomy). Following [63], [64], we investigated if any single morphologically identified species included multiple molecular lineages separated by a mean K2P distance greater than 2%. This criterion was first developed by [63], [64] to uncover hidden biodiversity within mammals (and especially bats) adopting a genetic species concept. Although this approach was initially based on the analysis of variation in the mitochondrial *cyt b*, some recent works reprised the assumptions of [63], [64] transposing them to the study of the variability in the barcode marker *coxI* among Neotropical and Southeast Asian bats populations [19], [38], [65].

We generated a neighbour-joining (NJ) phenetic tree based on comprehensive dataset in MEGA 4.0 [60]. The options used were: tree inference method: neighbour-joining; phylogeny test and options: bootstrap (1000 replicates); gaps/missing data: pairwise deletion; codon positions: 1st +2nd +3rd + non-coding; substitution model: K2P; substitutions to include: transitions + transversions; pattern among lineages: same (homogeneous); rates among sites: uniform rates. Although more sophisticated tree-building methods are available for deep branch resolution, we assumed that in a DNA barcoding context this approach was sufficient to resolve relationships at branch terminals.

No additional *coxI* sequences [30], [57] were available to improve the resolution in the *M. nattereri* complex. Therefore, to resolve known taxonomic inconsistencies in *M. nattereri* [30], [56], [57], we investigated the genetic structure of Italian *M. nattereri* and closely related congeners with two additional mitochondrial markers. These two molecular datasets (Table S2) included 21 *cyt b* sequences and 16 *ND1* sequences, of which, 19 and 10 respectively from previously published works [i.e. 30,57]. The sequences at these *loci* came from different individuals of Western Palearctic, so we treated the data as two distinct datasets instead of a single concatenated dataset. Based on the two datasets, we produced two NJ trees. Following [15], [63], [64], we used a NJ clustering method to identify different lineages and to flag potentially cryptic taxa that had mean K2P distances >2.0% with a bootstrap support greater than 95%. *M. myotis* ND1 (GenBank DQ120800) and *cyt b* (GenBank: AF246241) were used as the outgroups.

## Results

### Alignment Characteristics and DNA Barcoding Datasets

We amplified *coxI* fragments from all 209 samples and due to sequencing problems for oldest samples, we trimmed our barcode sequences to the same final length of 556 bp. No sequence contained insertion/deletions (indels), stop codons, or were biased by NUMT interference. Alignment analysis revealed average base frequencies as π_A_ = 0.256, π_C_ = 0.250, π_G_ = 0.166 and π_T_ = 0.328. The reference dataset included 182 *coxI* sequences (168 sequenced in this study and 14 from GenBank) that belonged to 30 of 34 Italian bat species from ten genera and four families (Table S1). For 23 echolocating bat species, the *coxI* sequences produced in this study were the first barcode entries ever deposited in GenBank. The average number of barcoded specimens per species was 5.84 (standard deviation = 4.44; range: 1–22). The minimum cumulative error, MCE (0.08%) occurred at OT = 4.4% (Figure S2). As shown in K2P distance graph (Figure S1), using the OT no overlap of intraspecific and interspecific nucleotide K2P distance occurred at values greater than the threshold, therefore excluding the presence of type I errors. By contrast, because some interspecific divergences were as low as 0%, type II errors [16] occurred when interspecific K2P distance was less than the OT.

Using this DNA barcoding method, we grouped samples nominally from the same species into coherent units for all but four taxa in the reference dataset (Figure S3). These four taxa were two pairs of closely related taxa that had observable morphological differences but had mean K2P distances lower than OT (*M. myotis* and *M. blythii* mean K2P distance = 1.56±0.31%; *Eptesicus serotinus* and *E. nilsonii* mean K2P distance = 0.91±0.38%). These type II errors caused the total cumulative error at the chosen threshold (i.e. OT) and led to the inclusion of these pairs of species into the same MOTUs. Among vespertilionids, average interspecific K2P distances were greater than OT. This allowed to successfully discriminating between the most problematic cryptic species pairs (i.e. 7.63% between *Pipistrellus pipistrellus* and *P. pygmaeus*, 14.90% between *Plecotus auritus* and *P. macrobullaris*, and 15.82% between *M. mystacinus* and *M. alcathoe*).

Considering all the *coxI* sequences obtained for the 31 Italian bats species investigated in this study, the mean±standard error K2P distance within a species was 0.44±0.78% (range: 0%–9.61%) and the mean±standard error K2P distance between species was 21.20±3.53% (range: 0%–28.64%). The overall mean diversity was 19.46±1.36%. Most species had low levels of intraspecific molecular diversity (<2%). By contrast, high intraspecific diversity levels observed in morphological-identified *M.* *nattereri* were the result of three divergent molecular lineages. This high diversity supports the exclusion of *M. nattereri* from the calculation of OT.

### Identification of Unclassified Samples through DNA Barcoding

Using the BOLD-IDS tool on the comprehensive dataset, 14 of 41 (34.1%) morphologically unidentified samples were successfully assigned to a known species (Table 1). All *Plecotus* samples were assigned to *P. auritus* or *P. macrobullaris*. By contrast, only 4 of 17 *Myotis* and 3 of 17 *Pipistrellus* samples were assigned to a species. The remaining 27 samples returned similarity matches higher than 99% with more than one species, thus the system cannot provide a clear specific assignment.

**Table 1:** BOLD-IDS and OT identification of unknown samples.

Voucher	Field identification	OT identification	BOLD identification	Database BOLD scores
MIB:ZPL:01211	*Myotis* sp.	*mystacinus*	*mystacinus* - cf. *aurascens*	99.64 - 99.46
MIB:ZPL:01214	*Myotis* sp.	*mystacinus*	*mystacinus* - cf. *aurascens*	99.64 - 99.46
MIB:ZPL:01216	*Myotis* sp.	*mystacinus*	*mystacinus* - cf. *aurascens*	99.64 - 99.46
MIB:ZPL:01221	*Myotis* sp.	*mystacinus*	*mystacinus* - cf. *aurascens*	99.64 - 99.46
MIB:ZPL:01222	*Myotis* sp.	*mystacinus*	*mystacinus* - cf. *aurascens*	99.64 - 99.46
MIB:ZPL:01223	*Myotis* sp.	*mystacinus*	*mystacinus* - cf. *aurascens*	99.64 - 99.46
MIB:ZPL:01228	*Myotis* sp.	*mystacinus*	cf. *aurascens* - *mystacinus*	99.64 - 99.46
MIB:ZPL:01230	*Myotis* sp.	*alcathoe*	*alcathoe*	99.82
MIB:ZPL:01235	*Myotis* sp.	*mystacinus*	cf. *aurascens* - *mystacinus*	99.64 - 99.46
MIB:ZPL:01281	*Myotis* sp.	*alcathoe*	*alcathoe*	100
MIB:ZPL:01287	*Myotis* sp.	*alcathoe*	*alcathoe*	100
MIB:ZPL:01289	*Myotis* sp.	*alcathoe*	*alcathoe*	100
MIB:ZPL:01301	*Myotis* sp.	*mystacinus*	*mystacinus* - cf. *aurascens*	99.64 - 99.46
MIB:ZPL:01302	*Myotis* sp.	*mystacinus*	*mystacinus* - cf. *aurascens*	99.64 - 99.46
MIB:ZPL:01303	*Myotis* sp.	*mystacinus*	*mystacinus* - cf. *aurascens*	99.64 - 99.46
MIB:ZPL:01256	*Myotis* sp.	*mystacinus*	*mystacinus* - cf. *aurascens*	99.64 - 99.46
MIB:ZPL:01319	*Myotis* sp.	*mystacinus*	*mystacinus* - cf. *aurascens*	99.64 - 99.46
MIB:ZPL:01239	*Pipistrellus* sp.	*pipistrellus*	*pipistrellus*	99.64
MIB:ZPL:01241	*Pipistrellus* sp.	*pipistrellus*	*pipistrellus*	99.64
MIB:ZPL:02288	*Pipistrellus* sp.	*pipistrellus*	*pipistrellus*	98.73
MIB:ZPL:03815	*Pipistrellus* sp.	*pygmaeus*	*pygmaeus* - *pipistrellus*	100 - 99.82
MIB:ZPL:03816	*Pipistrellus* sp.	*pygmaeus*	*pygmaeus* - *pipistrellus*	100 - 100
MIB:ZPL:03817	*Pipistrellus* sp.	*pygmaeus*	*pygmaeus* - *pipistrellus*	100 - 99.82
MIB:ZPL:03818	*Pipistrellus* sp.	*pygmaeus*	*pygmaeus* - *pipistrellus*	99.82 - 99.82
MIB:ZPL:03819	*Pipistrellus* sp.	*pygmaeus*	*pygmaeus* - *pipistrellus*	99.82 - 99.64
MIB:ZPL:03820	*Pipistrellus* sp.	*pygmaeus*	*pygmaeus* - *pipistrellus*	99.82 - 99.82
MIB:ZPL:03821	*Pipistrellus* sp.	*pygmaeus*	*pygmaeus* - *pipistrellus*	100 - 100
MIB:ZPL:03822	*Pipistrellus* sp.	*pygmaeus*	*pygmaeus* - *pipistrellus*	100 - 100
MIB:ZPL:03823	*Pipistrellus* sp.	*pygmaeus*	*pygmaeus* - *pipistrellus*	100 - 100
MIB:ZPL:03824	*Pipistrellus* sp.	*pygmaeus*	*pygmaeus* - *pipistrellus*	100 - 99.82
MIB:ZPL:03825	*Pipistrellus* sp.	*pygmaeus*	*pygmaeus* - *pipistrellus*	100 - 99.82
MIB:ZPL:03826	*Pipistrellus* sp.	*pygmaeus*	*pygmaeus* - *pipistrellus*	100 - 99.82
MIB:ZPL:03827	*Pipistrellus* sp.	*pygmaeus*	*pygmaeus* - *pipistrellus*	100 - 99.82
MIB:ZPL:03828	*Pipistrellus* sp.	*pygmaeus*	*pygmaeus* - *pipistrellus*	99.82 - 99.82
MIB:ZPL:03414	*Plecotus* sp.	*macrobullaris*	*macrobullaris*	99.82
MIB:ZPL:00262	*Plecotus* sp.	*macrobullaris*	*macrobullaris*	99.82
MIB:ZPL:01189	*Plecotus* sp.	*macrobullaris*	*macrobullaris*	99.82
MIB:ZPL:00265	*Plecotus* sp.	*auritus*	*auritus*	99.64
MIB:ZPL:00268	*Plecotus* sp.	*auritus*	*auritus*	100
MIB:ZPL:00269	*Plecotus* sp.	*auritus*	*auritus*	100
MIB:ZPL:00270	*Plecotus* sp.	*auritus*	*auritus*	100

List of identification results for 41 unrecognized bats sampled in Italy. Identification was performed by the IDS (identification engine on BOLD System [12]) and OT [55] approaches. Identity score and indecision cases returned by IDS are reported for each sample.

Using our reference dataset as a comparison, all 41 morphologically unidentified bats were unequivocally assigned to a known species. In all cases, each queried barcode sequence showed values of K2P distance lower than OT with each corresponding species included in the reference dataset (Table 1). Morphologically unrecognized samples were assigned to six cryptic species: *M.* *mystacinus*, *M. alcathoe*, *Plecotus auritus*, *P. macrobullaris*, *Pipistrellus pygmaeus* and *P. pipistrellus* (Figure S3). All assignments agreed with those from the BOLD-IDS tool.

### Detection of New Cryptic Lineages and Assignation to Intermediate Taxonomic Categories

**Figure 2:**
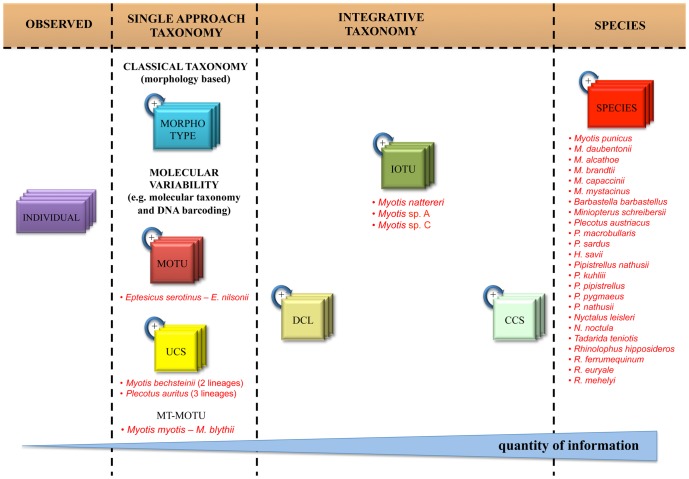
Subdivision of echolocating bats in the different taxonomic ranks. How to properly call all the different entities identified in our work of integrated taxonomy on Italian echolocating bats. It is important to observe the raise of information content proceeding from left to right.

Based on DNA barcoding data, morphological data, and preliminary data on geographic structure, we classified samples into ranks according to the information content of the groupings (Figure 2). For the 31 bat species in the comprehensive dataset, the OT cut-off revealed 31 molecular entities: 26 corresponding to morphologically assigned species, two MOTUs that each included two species, and three IOTUs that came from a single nominal species (Figure S3). Each of the two MOTUs was comprised of a pair of morphologically distinct species (*E. serotinus*–*E. nilsonii* and *M. myotis*–*M. blythii*). The three IOTUS were three divergent molecular lineages in *M. nattereri*.

In addition, at least 5 of the 31 morphologically identified species had multiple molecular lineages that had mean K2P distances >2% with high bootstrap support in the NJ reconstruction (Table 2; Figure S3).

**Table 2:** Divergent intraspecific molecular lineages.

Scientific name	Number of lineages	Geographical localization of the lineages	% Mean divergence	Bootstrap valuesbetween lineages
*Myotis myotis*	2	(NIT, CIT, SIT); (NIT)	3.51	100/99
*Myotis blythii*	2	(NIT, CIT, SIT); (NIT)	3.40	100/99
*Myotis nattereri*	3	(UK); (NIT); (CIT, SIT)	9.47, 9.34, 5.60	93/100/100
*Myotis bechsteinii*	2	(SI); (FR, NIT, CIT)	2.52	99/100
*Plecotus auritus*	3	(NIT, CIT); (NIT); (CIT, SIT)	2.52, 2.56, 2.62	100/98/91

List of Italian bats species with mean sequence divergence (K2P) between lineages greater than 2%. Locality group (NIT: Northern Italy; CIT: Central Italy; SIT: Southern Italy; UK: United Kingdom; FR: France) and bootstrap support (1000 replicates) for each lineage are also provided.

In particular, for the lineages in *Plecotus auritus* and *M. bechesteinii*, mean K2P distances higher than 2% but lower than OT (calculated on our reference dataset) and lower than 5% (i.e. the cut-off suggested by [64] as an indicative value for the occurrence of cryptic species), might suggest the presence of geographic structure, though no morphological variation was detected in the field with respect to biometric ranges for European populations published in [35] (data not shown). Given these assumptions, the variability showed by these two species clearly falls into the definition of UCS.

The DNA barcode sequences of *M. myotis* and *M. blythii*, which made up a single MOTU, could be divided into two molecular clusters separated by an average K2P distance of 3.51±0.77%. Both clusters contained samples from both morphological species (Figure S3; Table 2). Samples in one cluster were restricted to Northern Italy. The other cluster included individuals from all regions in Italy, including collection sites in the north of the peninsula where also the first lineage was observed (Table S1 and Figure S3).

*M. nattereri* samples belonged to three distinct *coxI* lineages with mean K2P distances greater than 2%, 5% and even OT (Figure S3 and Table 2). Two lineages had distinct geographic origins: one exclusively from northern Italy and the other from central and southern Italy. This congruence of molecular and biogeographic data designates them as IOTUs. This observed genetic structure was not paralleled by morphology, as samples from the lineages were morphologically similar. The average variability within these lineages was very low: 0.34±0.14% and 0.40±0.15% for the northern and central-southern lineages, respectively. The third lineage came from a single DNA barcode sequence from an English sample found in GenBank (GU270561). In addition to *coxI*, we investigated *M. nattereri* structure from *cyt b* (21 sequences, 768 bp long) and ND1 (17 sequences, 605 bp long) mtDNA genes. NJ trees from these datasets revealed five major lineages: one in North Africa (*Myotis* sp. B) and four in Europe (*M. nattereri* sensu stricto, *M. escalerai, Myotis* sp. A, and *Myotis* sp. C). All groups diverged for mean K2P distances of at least 7.8% and 7.6% for ND1 and *cyt b,* respectively (Figure S4). Again, these values are consistently higher than the limits proposed by [63], [64] to identify cryptic lineages in mammals deserving the rank of species if further details are provided. In this context, a clear geographic distinction of the lineages contributes to confirm this hypothesis. Northern Italian samples belonged to the lineage that also included haplotypes from northern Iberia and Austria (*Myotis* sp. A). Both ND1 and *cyt b* trees suggested that samples from central and southern Italy represent a previously undescribed lineage (*Myotis* sp. C). Both Italian lineages are consistently different from samples belonging to *M. nattereri* sensu stricto (*M. nattereri* in Figure S4), which was also observed in the *coxI* dataset (i.e. the specimen from UK; Figure S3). Finally, all European lineages were distinct from the North African lineage (*Myotis* sp. B) and the recently diverged *M. escalerai* (Figure S4).

## Discussion

DNA barcoding and other molecular methods are well-known powerful tools for identifying morphologically ambiguous taxa and revealing cryptic lineages within morphologically uniform taxa. However, the utility of DNA barcoding within molecular taxonomy remains controversial, and the debate is still open concerning the taxonomic value of the identified entities (see for example [66]–[68]). In an attempt to clarify molecular classification schemes and eliminate confusion in specimen identification, we introduced the concept of IOTU and tested its utility in the molecular taxonomy of Italian echolocating bats.

### Species Boundaries in Italian Bats

Our analyses provided strong support for a role for DNA barcoding in integrative taxonomy for recognizing molecular and biological entities. The low Minimum Cumulative Error (MCE) in the calculation of OT suggested a strong agreement between morphological identification and *coxI* molecular variability in the reference dataset, supporting the taxonomic value of IOTUs. For *M. nattereri*, the correlation between genetic variation and geographic origin of the samples defined three distinct IOTUs.

Our molecular dataset included samples that belonged to cryptic and/or recently described species (*M. brandtii*, *M. alcathoe*, *Plecotus macrobullaris*, and *Pipistrellus pygmaeus*), whose presence, distribution, and population size in the Italian peninsula are still poorly understood. In this context, our OT/MCE-based approach was successful in flagging potentially taxonomic criticisms (e.g. species showing high molecular variability) and granted a high discrimination power even in the detection of cryptic taxa.

Our analyses revealed two instances when morphological and molecular characterizations were inconsistent. Two pairs of vespertilionid congeners (*E. serotinus*-*E. nilsonii* and *M. myotis-M. blythii*) were described as two MOTUs. Although clearly distinguishable at the morphological level (and sometimes at the ecological and/or physiological one), these species cannot be clearly identified based on *coxI* despite some patterns of molecular divergence.

The molecular similarity at the DNA barcode sequences of the *Eptesicus* species has been previously observed for other mitochondrial markers [31], [69], [70] (Figure S3). A recent hypothesis posits that *E. serotinus* populations from Russia might have maintained their original mitochondrial lineages, which were lost in western populations due to complete introgression of mtDNA of *E. nilssonii* [70]. Further analyses with nuclear markers could confirm this hypothesis.

Similarly, reduced interspecific mtDNA variability was reported for European populations of *M. myotis* and *M. blythii*, which are sympatric across a wide range of southern and central Europe [31], [69], [71], [72]. MtDNA haplotypes are frequently shared between *M. myotis* and *M. blythii* in sympatric areas, such as the Alps and northern Italy [41], [69]. In agreement with these studies, our DNA barcoding approach revealed no clear genetic segregation between individuals from these taxa, limiting the delineation of distinct taxonomic entities. MtDNA and nuclear microsatellites revealed that approximately 25% of *M. blythii* had introgressed genes of *M.* *myotis* origin, but less than 4% of the *M. myotis* bats had introgressed genes from *M. blythii* [72]. Thus, these two species exhibit ongoing asymmetric hybridization in sympatric regions, including northern Italy. This hybridization pattern suggests a progressive loss of the mitochondrial genome of *M. blythii* in Europe through a series of introgression events occurred during the recent colonization by *M. blythii* from Asia. In addition to known hybridization in northern Italy, our results suggest that these species can also hybridize in central and southern Italy. However, more extensive sampling and the use of nuclear markers are required to confirm the occurrence of hybridization in these regions.

Moreover, the two *coxI* lineages in this MOTU (Figure S3 and Table 2) did not correspond to morphology or sampling location. Instead, they represent distinct sympatric lineages, which were also found in the same colony (e.g. Onferno Natural Reserve in northern Italy). This pattern of molecular variability deserves to be further investigated, though some cases of divergent molecular mtDNA lineages have already been observed in *M. myotis* from Italian populations [27]. It is possible that the two lineages represent population structure from multiple refugia during the climatic fluctuations of Pleistocene and a similar phenomenon might have occurred for other taxa [27].

As a final remark, it should be considered that the two *coxI* lineages can be interpreted as two UCS in spite of their variability (see Table 2). However, due to the complex taxonomic situation of *M. myotis* and *M. blythii*, it seems better to call them, from the DNA barcoding point of view, a single MOTU. In addition, to be more precise and to anticipate one of the conclusions of this work, we propose to call these undefined cases as Multi Taxa - Molecular Operational Taxonomic Units (MT-MOTUs) (see below).

### Identification of Unclassified Samples

The two DNA barcoding approaches used to identify cryptic species (i.e. BOLD-IDS and the comparison with reference dataset) agreed for only 34% of the morphologically unidentified samples (Table 1). While the reference dataset allows identifying all morphologically unassigned samples, BOLD-IDS only identified *Plecotus* species, *M. alcathoe*, and *Pipistrellus pipistrellus*. *M.* *mystacinus* and *P. pygmaeus* were not unequivocally assigned because of taxonomic uncertainties or morphological misclassification of reference specimens in BOLD with congeners. All *Myotis* samples identified as *M. mystacinus* by comparison with the reference dataset were characterized as indecision cases by IDS, which returned a “*Myotis mystacinus* – *M.* cf. *aurascens*” response. *M.* *aurascens* was considered a geographical morph of *M. mystacinus* that has been proposed as a new species based on slight morphological and karyotypic differences [73]–[75]. However, mtDNA variation was not distinct between *M. aurascens* and *M. mystacinus*, leaving its taxonomic status unclear [32], [40], [69]. Misclassification of morphological reference specimens reflects the practical difficulties in the morphological recognition of cryptic species only known from molecular data, such as the case of *Pipistrellus pygmaeus* vs. *P.* *pipistrellus*.

On the whole, these contrasting results highlight that nowadays, if we are dealing with taxa rich in cryptic species such as echolocating bats, a dedicated reference database is the core step to reduce the influence of misidentification. Moreover, we believe that in a local context, where biogeographical forces drive to the differentiation of isolated populations and even new putative species (e.g. the Italian Peninsula), the development of a local reference molecular database permits more resolution to understand species presence and boundaries than any available general archive.

### Detection of New Lineages and New Cryptic Species

We detected substantial intraspecific variation in DNA barcodes from five vespertilionid species that are widely distributed across Italy. Each species had two or three intraspecific *coxI* lineages with mean K2P genetic distances >2%. According to our scheme, some of these lineages can be tentatively considered as UCS. Similarly, if we adopt the criteria suggested by [63], [64], these lineages would be flagged as potentially containing cryptic species requiring additional taxonomic investigations. It should be also considered that *cyt b* (used to assess the 2% threshold as in [63], [64]) evolves at a faster rate than *coxI* [76]. Thus, as suggested by [65] the range of application of the criteria cited above might be properly resized when applied in a metazoan DNA barcoding context based on *coxI*.

In several cases, multiple intraspecific lineages occurred in syntopy (at both regional and site scale; see for example the case of *Plecotus auritus*) without any pattern of morphological differentiation. While introgression explains these intraspecific patterns in *M.* *blythii* [72], intraspecific variation in *Plecotus auritus* and *M. bechsteinii* cannot be explained so easily. *M. bechsteinii* might have experienced a population decline in the middle of the Neolithic (5000 years ago) as a result of habitat reduction. The fragmentation and isolation of small populations might have led to rapid population differentiation through drift therefore contributing to the molecular divergence of geographic lineages [77]. Preliminary biogeographic analyses [57], [78] showed contrasting patterns of intraspecific variation among European populations of *Plecotus* spp. and other vespertilionids. These studies highlighted the key role of the Iberian Peninsula as a refugium during the Pleistocene ice age, which led to divergence of cryptic lineages within some taxa. It is possible, that the Italian and Balkan peninsulas also acted as refugia, harbouring their own cryptic lineages [30], [57]. Comparisons with lineages sampled from other European locations might provide further evidence of the Italian peninsula as a glacial refugium. It should be acknowledged that the interpretation of divergent intraspecific mitochondrial lineages could not be trivial, especially if they are revealed using a single and female-inherited marker (e.g. *coxI*). As clearly demonstrated by [19] on an extensive survey of Neotropical bat populations, mitochondrial splits >2% can be the result of phylogeographic structuring as an effect of female philopatry or they can reflect the real occurrence of cryptic taxa. Both phenomena are equally plausible but to resolve the situation, the combined use of both mitochondrial and nuclear markers is desirable due to their different modes of inheritance.

We also detected a complex pattern of intraspecific molecular variation in *M. nattereri* sampled across Italy. Previous mtDNA and nuclear studies showed that the *M. nattereri* complex is paraphyletic and encompasses at least four different lineages distributed in the Palearctic (i.e. *M. nattereri*, *M. escalerai*, *Myotis* sp. A, *Myotis* sp. B) [30], [56], [57]. Two of these lineages, *M. nattereri* sensu stricto and *M.* *escalerai*, have been formally described based on morphological, ecological, and molecular characteristics. *M. nattereri* sensu stricto was first described by Kuhl (1817) and is distributed mainly in Central and Northern Europe. *M. escalerai* was first described by [58] but its taxonomic status as a distinct species was confirmed with molecular evidence [56], [57]. *M. escalerai* is distributed in the Iberian Peninsula and part of France. Our DNA barcoding data confirmed that *M. nattereri* from northern Italy were distinct from those from central and southern Italy and northern Europe (Table 2 and Figure S3). Analysis of other mitochondrial markers (ND1 and *cyt b*) clustered specimens from northern Italy with those from northern Iberia and the Alps (*Myotis* sp. A). *M. nattereri* samples from central and southern Italy formed a divergent lineage (*Myotis* sp. C) that had not been previously observed in the western Palearctic.

The high level of variation between these lineages is greater than the thresholds conventionally used to flag the occurrence of different species following the genetic species concept proposed by [63], [64] (i.e. >5% K2P). However, no preliminary descriptions or taxonomic synonyms have been given to any of these lineages, even the North African *Myotis* sp. B. Thus, the IOTU status for these lineages based on molecular divergence and biogeography could lead to a formal description of these entities once supplementary details (e.g. detailed morphological and/or ecological data) are provided. However, as well discussed by [19], [65], the use of mitochondrial markers only, even if coupled with geographical, ecological and other sources of data, does not necessarily associate with gene flow. This is a main problem in mammals where high male biased gene flow and female philopatry are common, especially for *Myotis* bats [79]. More extensive sampling across the entire Italian peninsula (and related islands) and the use of bi-parentally inherited markers could be used to assess the extent of gene flow between the two distinct lineages we observed for Italy.

### MOTUs, IOTUs and Taxonomy

Defining a biological species is not a simple matter. As dynamic, evolving entities, species are not unequivocally defined [80] and there are a variety of species concepts [81]. It is not trivial to determine which concept best fits samples classified by molecular data [63], [64]. In such complex situations, species designations based on a single category of taxonomic features (morphological, ecological, molecular, or biogeographic) is questionable. On the other hand, when multiple lines of evidence are available, it is unclear which data are most important in defining the species. Unfortunately, this is a serious limit because scientists usually deal with measurable values and reasonably controlled variables. Understanding how to balance the different data types will be important for classifying from multiple sources.

To standardize an integrated approach for taxonomy, we formally proposed a new entity, the Integrated Operational Taxonomic Unit (IOTU). This concept links different data sources in taxonomy, allowing morphological, ecological, geographical and other characteristics of living beings to be better combined with molecular data. IOTUs are defined by molecular lineages that have further support from at least one more part of the “taxonomic circle” [2] (Figure 1). The use of IOTUs should play a key role to shed light on the winding road towards species definitions. The results on our bat dataset showed this clearly: in the context of a taxonomic work a researcher is dealing, at the same time, with several kinds of biological entities that are “filling the gap” between individuals and species (Figure 2). As a matter of fact, it is often almost impossible to reach the level of species with a single approach, but not all the entities identified with molecular techniques are “simple” MOTUs, as apparently is thought in many DNA barcoding papers.

A final consideration: the term MOTU is used ambiguously in DNA barcoding literature. We propose here that it should only be used for its original definition only (i.e. M1 group, see introduction): “a group of undetermined organisms sharing a common molecular variability” [7], and we suggest alternatives for its other definitions. For MOTUs included in the M2 group (see introduction), we suggest that UCS is a better definition. For MOTUs included in the M3 group (see introduction), we suggest the designation Multi Taxa - Molecular Operational Taxonomic Units (MT-MOTUs), reflecting the low information content, which is often the result of the poor resolution of the molecular marker or of the pattern of evolution of organelle’s markers (such as the case of *M. myotis* and *M. blythii*).

Although divergent lineages do not always reflect distinct species or other taxonomic ranks, molecular data remains at the core of current taxonomic approaches. However, the future of taxonomy is not only in molecular markers. Rather, the future of modern taxonomy is more and more oriented towards the definition of the best way to integrate molecular data into multidisciplinary taxonomic approaches. In this context, the concept of IOTU is a major innovation for future taxonomic studies. The web of entities commented and described here (Figure 1) represents a ranking system that can improve the interpretation of data in integrated taxonomic approaches.

In applying this vision to the study of Italian bats, we showed that species, IOTUs, and even MOTUs, CCSs, UCSs, or DCLs yield information that can be meaningful to assess ecological requirements and/or conservation needs. In other words, all of these entities can be considered valuable conservation units. Thus, molecular identification techniques, such as DNA barcoding, play a major role in describing existing patterns of biodiversity, which are needed to design realistic actions for conservation management plans.

## Supporting Information

Figure S1 **Frequency distribution of intraspecific and interspecific genetic divergences in morphologically identified echolocating bats from Italy.** Graph shows intraspecific (yellow bars) and interspecific (red bars) comparisons across the bats species included in the reference dataset. Distances were calculated by MEGA 4.0 (pairwise deletion), using Kimura’s two-parameters substitution model.
(PDF)

Figure S2 **Cumulative error plot.** Minimum cumulative error analysis conducted on the reference dataset of Italian echolocating bats species. Type I (yellow) and type II (red) errors obtained with different thresholds.
(PDF)

Figure S3 **NJ reconstruction of Italian echolocating bats** ***coxI*** **sequences.** Neighbour joining tree based on *coxI* sequences of Italian echolocating bats generated with MEGA. Square brackets indicate the different taxonomic ranks corresponding to species, MOTUs (dotted line), and IOTUs (bold line) inferred by OT. As reported in Figure 2, *Myotis bechsteinii* and *Plecotus* auritus, should be assigned to the UCS rank. For each bat, voucher number and locality group are also provided (further details can be retrieved from Table S1). Bootstrap support (1000 replicates) values >70% are indicated above the nodes.
(PDF)

Figure S4 **NJ reconstructions of** ***Myotis nattereri*** **species complex based on** ***ND1*** **and** ***cyt b*** **sequences.** Phenetic relationships among sequences of the *cyt b* and *ND1* genes for Italian and European lineages belonging to the species complex *Myotis nattereri*. Locality groups are shown as follows: SIT, Southern Italy; CIT, Central Italy; SIT, Southern Italy; SMO, southern Morocco; CMO, central Morocco; NMO, northern Morocco; SIB, southern Iberia; NIB, northern Iberia; GER, Germany; AUS, Austria; SWI, Switzerland; GRE, Greece; HUN, Hungary. Bootstrap support (1000 replicates) values >70% are indicated above the nodes. Corresponding lineages are indicated by square brackets and named as reported in the manuscript. For further details about samples and owner of the sequences see Table S2.
(PDF)

Table S1 **List of biological samples, GenBank accessions and sampling details.** Bats examined in this study using a DNA barcoding approach with reference to specimen voucher (when available), family and species attribution (except for unrecognized bats), GenBank accession numbers, sampling localities (with province) and assigned locality group names (NIT: Northern Italy; CIT: Central Italy; SIT: Southern Italy; SAR: Sardinia; SW: Switzerland; IE: Ireland; FR: France; DE: Germany; UK: United Kingdom). Samples highlighted in bold have been included in the reference dataset and used for OT calculation. *cox*I sequences from GU270553 to GU270566 were retrieved in GenBank.
(PDF)

Table S2 **List of GenBank accession numbers and sampling details of bats belonging to the** ***M. nattereri*** **species complex.** Sampling locality details, accession numbers with related reference of *cyt b* and *ND1* sequences and name of corresponding lineage are provided for each individual.
(PDF)
