# Supplementary figures and images for: Integrated Operational Taxonomic Units (IOTUs) in Echolocating Bats: A Bridge between Molecular and Traditional Taxonomy
Source: PLoS One. 2012 Jun 28;7(6):e40122. doi: 10.1371/journal.pone.0040122 (PMC3386196; doi:10.1371/journal.pone.0040122)

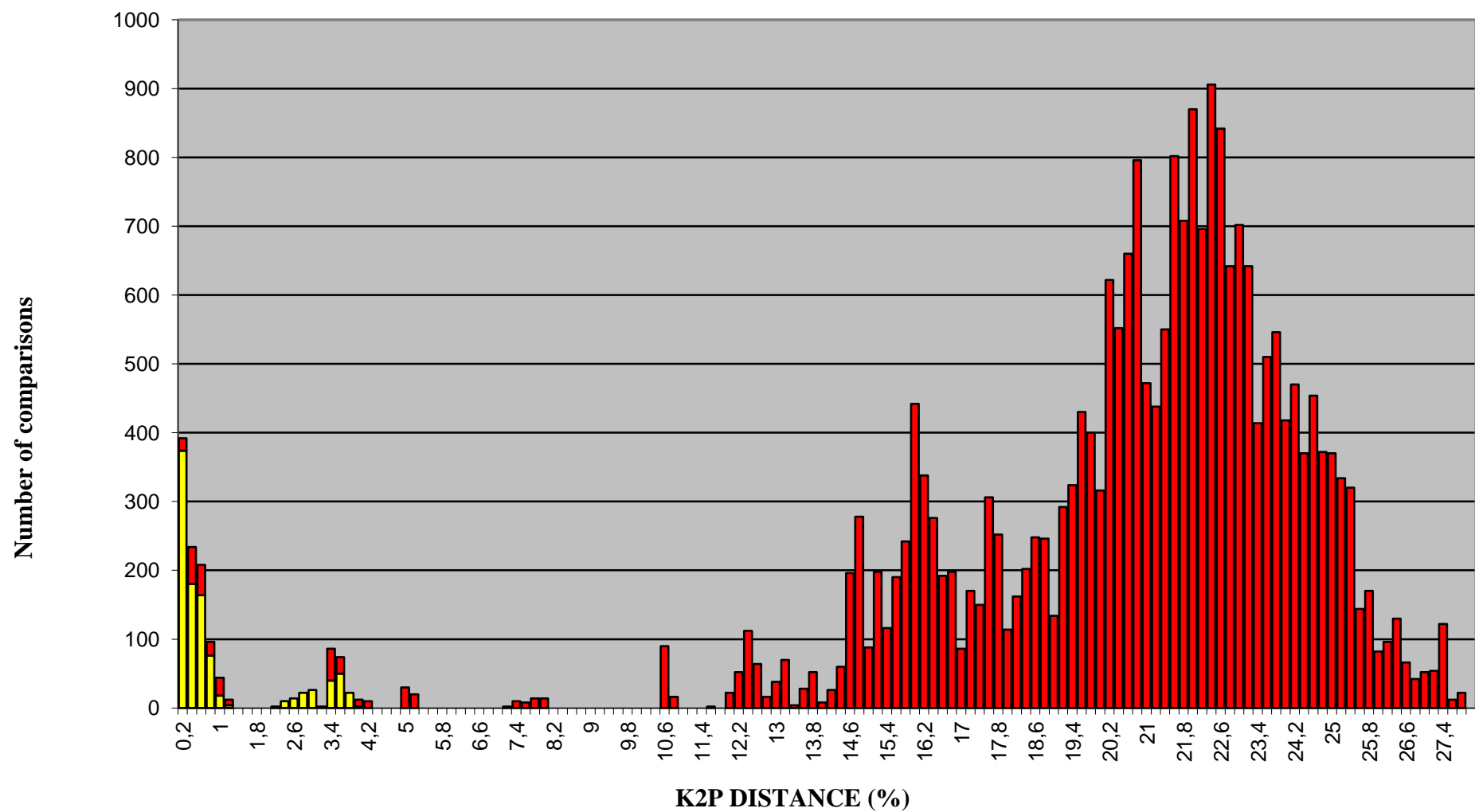

Supplement: Figure S1 — Frequency distribution of intraspecific and interspecific genetic divergences in morphologically identified echolocating bats from Italy. Graph shows intraspecific (yellow bars) and interspecific (red bars) comparisons across the bats species included in the reference dataset. Distances were calculated by MEGA 4.0 (pairwise deletion), using Kimura’s two-parameters substitution model. (PDF) [file pone.0040122.s001.pdf]

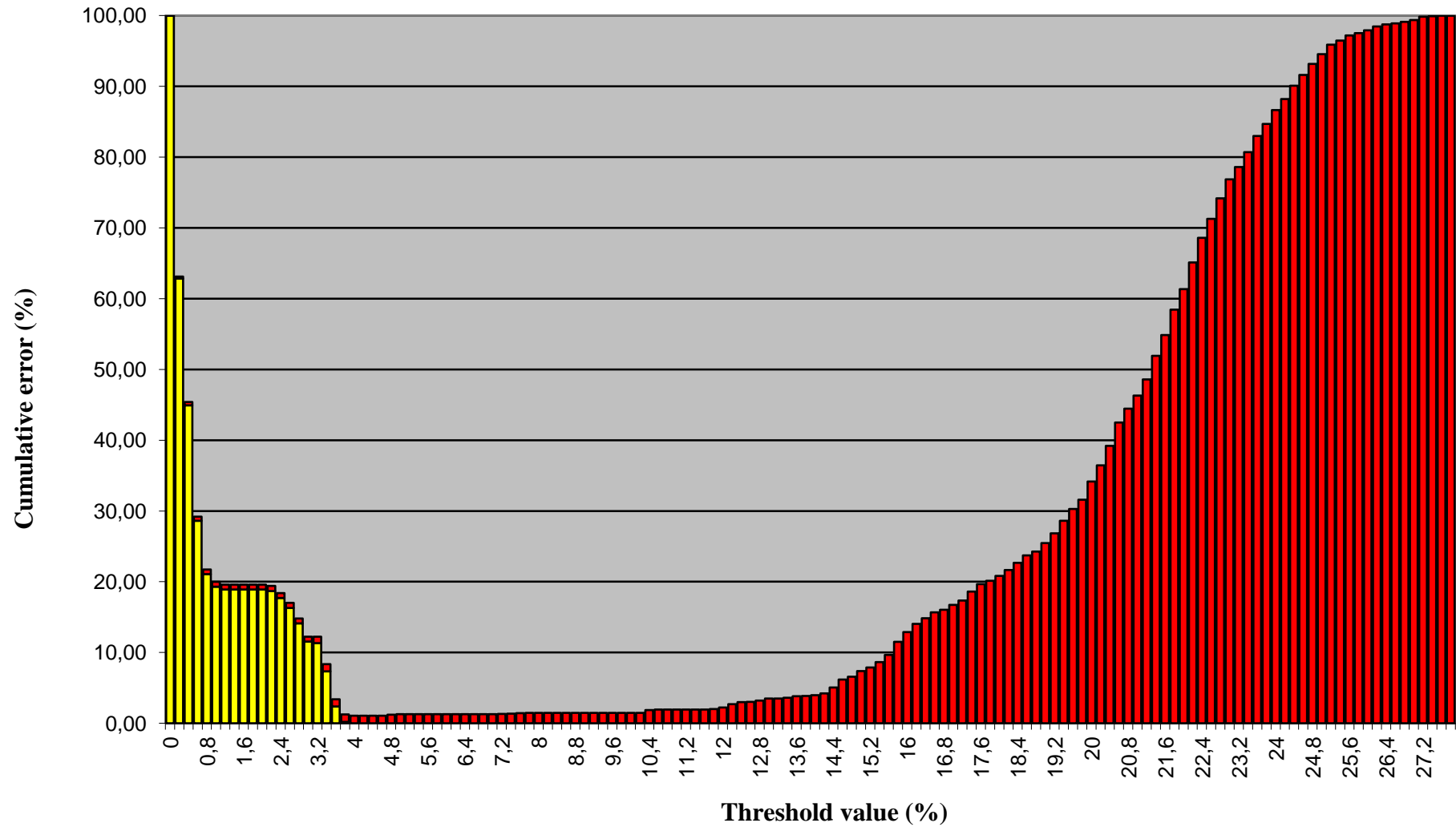

Supplement: Figure S2 — Cumulative error plot. Minimum cumulative error analysis conducted on the reference dataset of Italian echolocating bats species. Type I (yellow) and type II (red) errors obtained with different thresholds. (PDF) [file pone.0040122.s002.pdf]

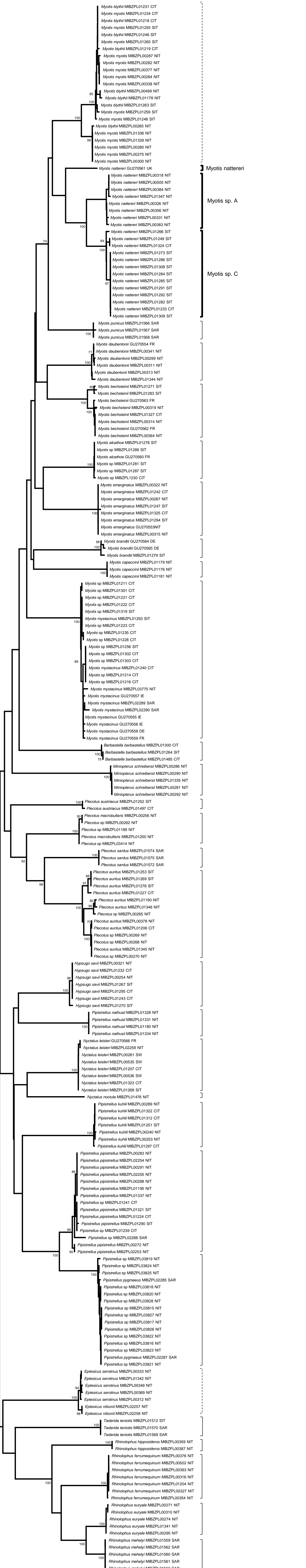

Supplement: Figure S3 — NJ reconstruction of Italian echolocating bats coxI sequences. Neighbour joining tree based on coxI sequences of Italian echolocating bats generated with MEGA. Square brackets indicate the different taxonomic ranks corresponding to species, MOTUs (dotted line), and IOTUs (bold line) inferred by OT. As reported in Figure 2, Myotis bechsteinii and Plecotus auritus, should be assigned to the UCS rank. For each bat, voucher number and locality group are also provided (further details can be retrieved from Table S1). Bootstrap support (1000 replicates) values >70% are indicated above the nodes. (PDF) [file pone.0040122.s003.pdf]

a) *cyt b*

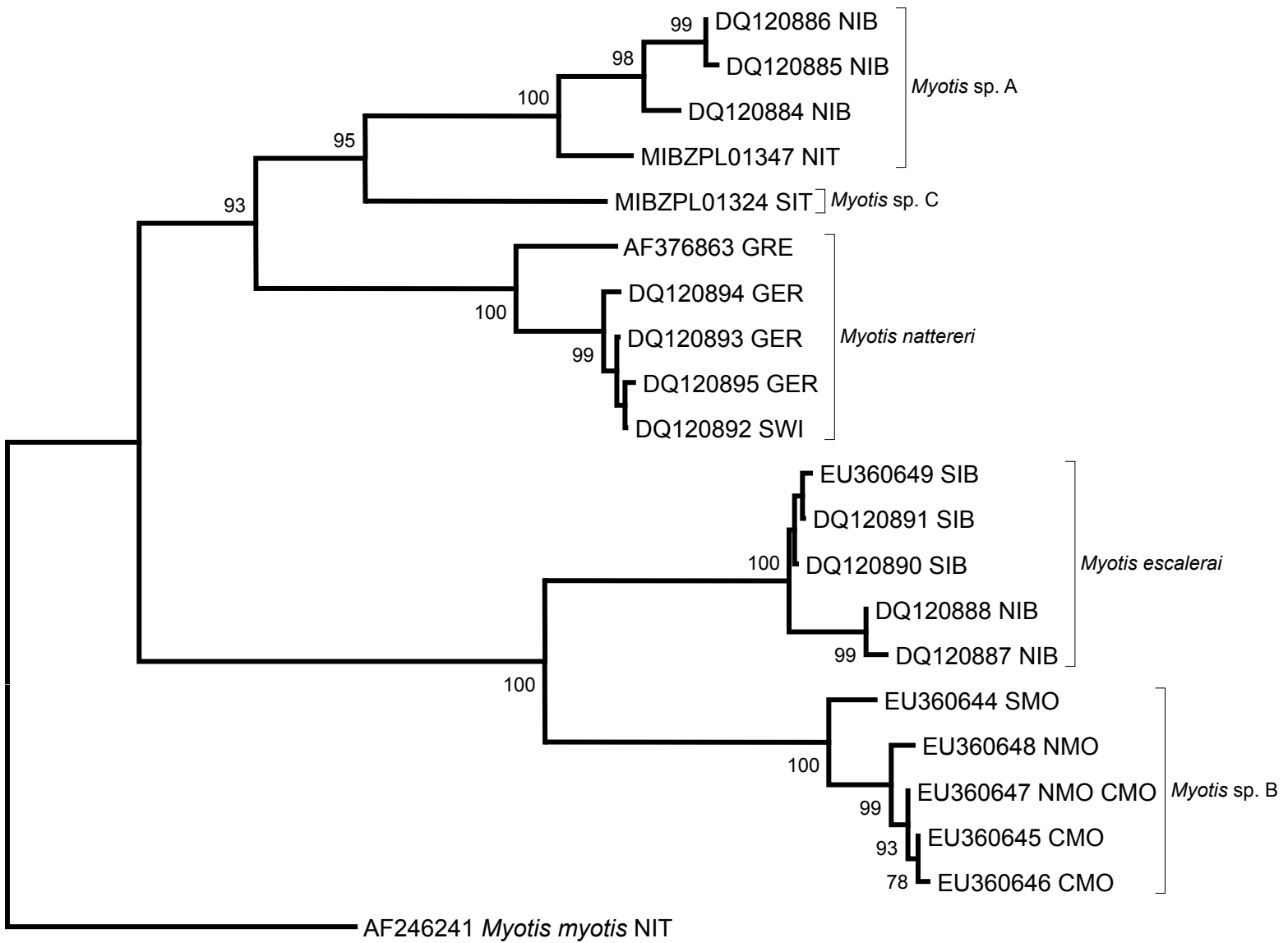

0.02

b) *ND1*

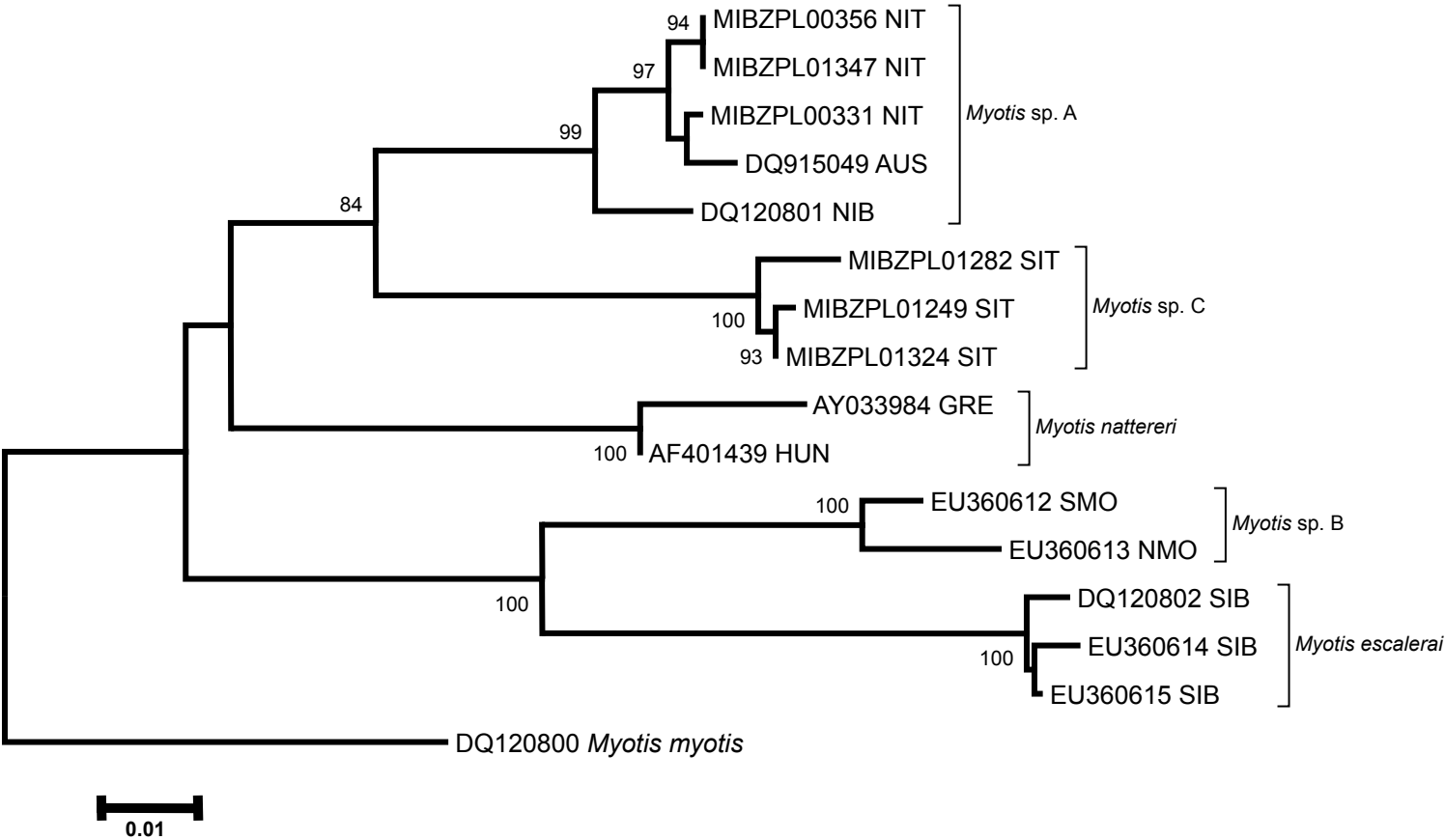

Supplement: Figure S4 — NJ reconstructions of Myotis nattereri species complex based on ND1 and cyt b sequences. Phenetic relationships among sequences of the cyt b and ND1 genes for Italian and European lineages belonging to the species complex Myotis nattereri. Locality groups are shown as follows: SIT, Southern Italy; CIT, Central Italy; SIT, Southern Italy; SMO, southern Morocco; CMO, central Morocco; NMO, northern Morocco; SIB, southern Iberia; NIB, northern Iberia; GER, Germany; AUS, Austria; SWI, Switzerland; GRE, Greece; HUN, Hungary. Bootstrap support (1000 replicates) values >70% are indicated above the nodes. Corresponding lineages are indicated by square brackets and named as reported in the manuscript. For further details about samples and owner of the sequences see Table S2. (PDF) [file pone.0040122.s004.pdf]
