# Supplementary material for: Integrated Operational Taxonomic Units (IOTUs) in Echolocating Bats: A Bridge between Molecular and Traditional Taxonomy
Source: PLoS One. 2012 Jun 28;7(6):e40122. doi: 10.1371/journal.pone.0040122 (PMC3386196; doi:10.1371/journal.pone.0040122)
Supplement: Table S1 — List of biological samples, GenBank accessions and sampling details. Bats examined in this study using a DNA barcoding approach with reference to specimen voucher (when available), family and species attribution (except for unrecognized bats), GenBank accession numbers, sampling localities (with province) and assigned locality group names (NIT: Northern Italy; CIT: Central Italy; SIT: Southern Italy; SAR: Sardinia; SW: Switzerland; IE: Ireland; FR: France; DE: Germany; UK: United Kingdom). Samples highlighted in bold have been included in the reference dataset and used for OT calculation. coxI sequences from GU270553 to GU270566 were retrieved in GenBank. (PDF) [file pone.0040122.s005.pdf]

Voucher	Sample name	Species	Family	GenBank Accession Nos.	Locality	Locality group
**MIB:ZPL:00281**	1707	*Miniopterus schreibersii* (Kuhl, 1817)	Miniopteridae	**FR856655**	Onferno Natural Reserve (RN)	NIT
**MIB:ZPL:00286**	1407	*Miniopterus schreibersii* (Kuhl, 1817)	Miniopteridae	**FR856656**	Onferno Natural Reserve (RN)	NIT
**MIB:ZPL:00290**	2007	*Miniopterus schreibersii* (Kuhl, 1817)	Miniopteridae	**FR856657**	Onferno Natural Reserve (RN)	NIT
**MIB:ZPL:00292**	1607	*Miniopterus schreibersii* (Kuhl, 1817)	Miniopteridae	**FR856658**	Onferno Natural Reserve (RN)	NIT
**MIB:ZPL:01335**	1307	*Miniopterus schreibersii* (Kuhl, 1817)	Miniopteridae	**FR856659**	Onferno Natural Reserve (RN)	NIT
**MIB:ZPL:01512**	MZUF 18577	*Tadarida teniotis* (Rafinesque, 1814)	Molossidae	**FR856844**	Pizzo, Calabria (VV)	SIT
**MIB:ZPL:01569**	210	*Tadarida teniotis* (Rafinesque, 1814)	Molossidae	**FR856845**	Sardinia	SAR
**MIB:ZPL:01570**	211	*Tadarida teniotis* (Rafinesque, 1814)	Molossidae	**FR856846**	Sardinia	SAR
**MIB:ZPL:00271**	2007	*Rhinolophus euryale* Blasius, 1853	Rhinolophidae	**FR856825**	Onferno Natural Reserve (RN)	NIT
**MIB:ZPL:00274**	2707	*Rhinolophus euryale* Blasius, 1853	Rhinolophidae	**FR856826**	Onferno Natural Reserve (RN)	NIT
**MIB:ZPL:00295**	1807	*Rhinolophus euryale* Blasius, 1853	Rhinolophidae	**FR856827**	Onferno Natural Reserve (RN)	NIT
**MIB:ZPL:00310**	2507	*Rhinolophus euryale* Blasius, 1853	Rhinolophidae	**FR856828**	Onferno Natural Reserve (RN)	NIT
**MIB:ZPL:01341**	1907	*Rhinolophus euryale* Blasius, 1853	Rhinolophidae	**FR856829**	Onferno Natural Reserve (RN)	NIT
**MIB:ZPL:00502**	0008	*Rhinolophus ferrumequinum* (Schreber, 1774)	Rhinolophidae	**FR856835**	Prevalle (BS)	NIT
**MIB:ZPL:00316**	0307	*Rhinolophus ferrumequinum* (Schreber, 1774)	Rhinolophidae	**FR856830**	Onferno Natural Reserve (RN)	NIT
**MIB:ZPL:00354**	_	*Rhinolophus ferrumequinum* (Schreber, 1774)	Rhinolophidae	**FR856832**	Onferno Natural Reserve (RN)	NIT
**MIB:ZPL:00376**	0207	*Rhinolophus ferrumequinum* (Schreber, 1774)	Rhinolophidae	**FR856833**	Onferno Natural Reserve (RN)	NIT
**MIB:ZPL:00393**	0107	*Rhinolophus ferrumequinum* (Schreber, 1774)	Rhinolophidae	**FR856834**	Onferno Natural Reserve (RN)	NIT
**MIB:ZPL:01204**	05	*Rhinolophus ferrumequinum* (Schreber, 1774)	Rhinolophidae	**FR856836**	San Cesario (MO)	NIT
**MIB:ZPL:00327**	0007	*Rhinolophus ferrumequinum* (Schreber, 1774)	Rhinolophidae	**FR856831**	Saronno (VA)	NIT
**MIB:ZPL:00359**	1207	*Rhinolophus hipposideros* (Bechstein, 1800)	Rhinolophidae	**FR856837**	Onferno Natural Reserve (RN)	NIT
**MIB:ZPL:00387**	_	*Rhinolophus hipposideros* (Bechstein, 1800)	Rhinolophidae	**FR856838**	Onferno Natural Reserve (RN)	NIT
**MIB:ZPL:01559**	200	*Rhinolophus mehelyi* Matschie, 1901	Rhinolophidae	**FR856839**	Sardinia	SAR
**MIB:ZPL:01560**	201	*Rhinolophus mehelyi* Matschie, 1901	Rhinolophidae	**FR856840**	Sardinia	SAR
**MIB:ZPL:01561**	202	*Rhinolophus mehelyi* Matschie, 1901	Rhinolophidae	**FR856841**	Sardinia	SAR
**MIB:ZPL:01562**	203	*Rhinolophus mehelyi* Matschie, 1901	Rhinolophidae	**FR856842**	Sardinia	SAR
**MIB:ZPL:01563**	204	*Rhinolophus mehelyi* Matschie, 1901	Rhinolophidae	**FR856843**	Sardinia	SAR
**MIB:ZPL:01485**	MZUF 14662	*Barbastella barbastellus* (Schreber, 1774)	Vespertilionidae	**FR856640**	Firenze (FI)	CIT
**MIB:ZPL:01264**	BABA65B	*Barbastella barbastellus* (Schreber, 1774)	Vespertilionidae	**FR856638**	Sant'Angelo D'Alife (CE)	SIT
**MIB:ZPL:01300**	BABA155B	*Barbastella barbastellus* (Schreber, 1774)	Vespertilionidae	**FR856639**	Monte Navegna Natural Reserve (RI)	CIT
**MIB:ZPL:02257**	_	*Eptesicus nilsonii* (Keyserling & Blasius, 1839)	Vespertilionidae	**FR856641**	Chiareggio (SO)	NIT
**MIB:ZPL:02258**	_	*Eptesicus nilsonii* (Keyserling & Blasius, 1839)	Vespertilionidae	**FR856642**	Chiareggio (SO)	NIT
**MIB:ZPL:00312**	0407	*Eptesicus serotinus* (Schreber, 1774)	Vespertilionidae	**FR856643**	Saronno (VA)	NIT
**MIB:ZPL:00333**	0807	*Eptesicus serotinus* (Schreber, 1774)	Vespertilionidae	**FR856644**	Saronno (VA)	NIT
**MIB:ZPL:00349**	0007	*Eptesicus serotinus* (Schreber, 1774)	Vespertilionidae	**FR856645**	Saronno (VA)	NIT
**MIB:ZPL:00369**	0207	*Eptesicus serotinus* (Schreber, 1774)	Vespertilionidae	**FR856646**	Saronno (VA)	NIT
**MIB:ZPL:01342**	0707	*Eptesicus serotinus* (Schreber, 1774)	Vespertilionidae	**FR856647**	Saronno (VA)	NIT
**MIB:ZPL:01232**	HYSA36B	*Hypsugo savii* (Bonaparte, 1837)	Vespertilionidae	**FR856650**	Villavallelonga (AQ)	CIT
**MIB:ZPL:00254**	_	*Hypsugo savii* (Bonaparte, 1837)	Vespertilionidae	**FR856648**	Parco del Campo dei Fiori (VA)	NIT
**MIB:ZPL:00321**	0207	*Hypsugo savii* (Bonaparte, 1837)	Vespertilionidae	**FR856649**	Cariadeghe (BS)	NIT
**MIB:ZPL:01267**	HYSA68B	*Hypsugo savii* (Bonaparte, 1837)	Vespertilionidae	**FR856652**	Corleto Monforte (SA)	SIT
**MIB:ZPL:01270**	HYSA71B	*Hypsugo savii* (Bonaparte, 1837)	Vespertilionidae	**FR856653**	Corleto Monforte (SA)	SIT
**MIB:ZPL:01243**	HYSA47	*Hypsugo savii* (Bonaparte, 1837)	Vespertilionidae	**FR856651**	Duchessa (RI)	CIT

Voucher	Sample name	Species	Family	GenBank Accession Nos.	Locality	Locality group
**MIB:ZPL:01295**	HYSA150B	*Hypsugo savii* (Bonaparte, 1837)	Vespertilionidae	**FR856654**	Monte Navegna Natural Reserve (RI)	CIT
**_**	_	*Myotis alcathoe* von Helversen & Heller, 2001	Vespertilionidae	**GU270560**	_	FR
**MIB:ZPL:01278**	MYMYS79B	*Myotis alcathoe* von Helversen & Heller, 2001	Vespertilionidae	**FR856660**	Corleto Monforte (SA)	SIT
**_**	_	*Myotis bechsteinii* (Kuhl, 1817)	Vespertilionidae	**GU270562**	_	FR
**_**	_	*Myotis bechsteinii* (Kuhl, 1817)	Vespertilionidae	**GU270563**	_	FR
**MIB:ZPL:00314**	1707	*Myotis bechsteinii* (Kuhl, 1817)	Vespertilionidae	**FR856661**	Parco del Campo dei Fiori (VA)	NIT
**MIB:ZPL:00319**	1007	*Myotis bechsteinii* (Kuhl, 1817)	Vespertilionidae	**FR856662**	Parco del Campo dei Fiori (VA)	NIT
**MIB:ZPL:00364**	0107	*Myotis bechsteinii* (Kuhl, 1817)	Vespertilionidae	**FR856663**	Cariadeghe (BS)	NIT
**MIB:ZPL:01271**	MYBEC72B	*Myotis bechsteinii* (Kuhl, 1817)	Vespertilionidae	**FR856664**	Corleto Monforte (SA)	SIT
**MIB:ZPL:01283**	MYBECH84B	*Myotis bechsteinii* (Kuhl, 1817)	Vespertilionidae	**FR856665**	Felitto (SA)	SIT
**MIB:ZPL:01327**	UMMBE1B	*Myotis bechsteinii* (Kuhl, 1817)	Vespertilionidae	**FR856666**	Umbria	CIT
**MIB:ZPL:01218**	MYBLY22B	*Myotis blythii* (Tomes, 1857)	Vespertilionidae	**FR856670**	Villavallelonga (AQ)	CIT
**MIB:ZPL:01219**	MYBLY23B	*Myotis blythii* (Tomes, 1857)	Vespertilionidae	**FR856671**	Villavallelonga (AQ)	CIT
**MIB:ZPL:01231**	MYBLY35	*Myotis blythii* (Tomes, 1857)	Vespertilionidae	**FR856672**	Villavallelonga (AQ)	CIT
**MIB:ZPL:00499**	0008	*Myotis blythii* (Tomes, 1857)	Vespertilionidae	**FR856668**	Prevalle (BS)	NIT
**MIB:ZPL:01178**	0108	*Myotis blythii* (Tomes, 1857)	Vespertilionidae	**FR856669**	Prevalle (BS)	NIT
**MIB:ZPL:01246**	MYBLY50B	*Myotis blythii* (Tomes, 1857)	Vespertilionidae	**FR856673**	Cusano Mutri (CE)	SIT
**MIB:ZPL:01263**	MYMYO64B	*Myotis blythii* (Tomes, 1857)	Vespertilionidae	**FR856674**	Sant'Angelo D'Alife (CE)	SIT
**MIB:ZPL:00285**	0007	*Myotis blythii* (Tomes, 1857)	Vespertilionidae	**FR856667**	Onferno Natural Reserve (RN)	NIT
**_**	_	*Myotis brandtii* (Eversmann, 1845)	Vespertilionidae	**GU270564**	_	DE
**_**	_	*Myotis brandtii* (Eversmann, 1845)	Vespertilionidae	**GU270565**	_	DE
**MIB:ZPL:01279**	MYBRA80B	*Myotis brandtii* (Eversmann, 1845)	Vespertilionidae	**FR856675**	Corleto Monforte (SA)	SIT
**MIB:ZPL:01176**	0108	*Myotis capaccinii* (Bonaparte, 1837)	Vespertilionidae	**FR856676**	Prevalle (BS)	NIT
**MIB:ZPL:01179**	0008	*Myotis capaccinii* (Bonaparte, 1837)	Vespertilionidae	**FR856677**	Prevalle (BS)	NIT
**MIB:ZPL:01181**	0208	*Myotis capaccinii* (Bonaparte, 1837)	Vespertilionidae	**FR856678**	Prevalle (BS)	NIT
**_**	_	*Myotis daubentonii* (Kuhl, 1817)	Vespertilionidae	**GU270554**	_	FR
**MIB:ZPL:00341**	1407	*Myotis daubentonii* (Kuhl, 1817)	Vespertilionidae	**FR856682**	Parco del Campo dei Fiori (VA)	NIT
**MIB:ZPL:01344**	1107	*Myotis daubentonii* (Kuhl, 1817)	Vespertilionidae	**FR856683**	Parco del Campo dei Fiori (VA)	NIT
**MIB:ZPL:00311**	1607	*Myotis daubentonii* (Kuhl, 1817)	Vespertilionidae	**FR856680**	Cariadeghe (BS)	NIT
**MIB:ZPL:00313**	0707	*Myotis daubentonii* (Kuhl, 1817)	Vespertilionidae	**FR856681**	Cariadeghe (BS)	NIT
**MIB:ZPL:00299**	0007	*Myotis daubentonii* (Kuhl, 1817)	Vespertilionidae	**FR856679**	Valganna (VA)	NIT
**_**	_	*Myotis emarginatus* (Geoffroy, 1806)	Vespertilionidae	**GU270553**	_	NIT
**MIB:ZPL:00267**	_	*Myotis emarginatus* (Geoffroy, 1806)	Vespertilionidae	**FR856684**	Parco del Campo dei Fiori (VA)	NIT
**MIB:ZPL:00315**	1207	*Myotis emarginatus* (Geoffroy, 1806)	Vespertilionidae	**FR856685**	Cariadeghe (BS)	NIT
**MIB:ZPL:00322**	0907	*Myotis emarginatus* (Geoffroy, 1806)	Vespertilionidae	**FR856686**	Cariadeghe (BS)	NIT
**MIB:ZPL:01294**	MYEM97B	*Myotis emarginatus* (Geoffroy, 1806)	Vespertilionidae	**FR856689**	Felitto (SA)	SIT
**MIB:ZPL:01242**	MYEM46B	*Myotis emarginatus* (Geoffroy, 1806)	Vespertilionidae	**FR856687**	Borgorose (RI)	CIT
**MIB:ZPL:01247**	MYEM51B	*Myotis emarginatus* (Geoffroy, 1806)	Vespertilionidae	**FR856688**	Cusano Mutri (CE)	SIT
**MIB:ZPL:01325**	UMMEM1B	*Myotis emarginatus* (Geoffroy, 1806)	Vespertilionidae	**FR856690**	Umbria	CIT
**MIB:ZPL:01234**	MYMYO38	*Myotis myotis* (Borkhausen, 1797)	Vespertilionidae	**FR856699**	Villavallelonga (AQ)	CIT
**MIB:ZPL:01248**	MYMYO52B	*Myotis myotis* (Borkhausen, 1797)	Vespertilionidae	**FR856700**	Cusano Mutri (CE)	SIT
**MIB:ZPL:01255**	MYMYO58B	*Myotis myotis* (Borkhausen, 1797)	Vespertilionidae	**FR856701**	Cusano Mutri (CE)	SIT
**MIB:ZPL:01259**	MYMYO60B	*Myotis myotis* (Borkhausen, 1797)	Vespertilionidae	**FR856702**	Cusano Mutri (CE)	SIT

Voucher	Sample name	Species	Family	GenBank Accession Nos.	Locality	Locality group
**MIB:ZPL:01265**	MYMYO66B	*Myotis myotis* (Borkhausen, 1797)	Vespertilionidae	**FR856703**	Sant'Angelo D'Alife (CE)	SIT
**MIB:ZPL:00275**	2707	*Myotis myotis* (Borkhausen, 1797)	Vespertilionidae	**FR856691**	Onferno Natural Reserve (RN)	NIT
**MIB:ZPL:00280**	2507	*Myotis myotis* (Borkhausen, 1797)	Vespertilionidae	**FR856692**	Onferno Natural Reserve (RN)	NIT
**MIB:ZPL:00282**	2607	*Myotis myotis* (Borkhausen, 1797)	Vespertilionidae	**FR856693**	Onferno Natural Reserve (RN)	NIT
**MIB:ZPL:00284**	2907	*Myotis myotis* (Borkhausen, 1797)	Vespertilionidae	**FR856694**	Onferno Natural Reserve (RN)	NIT
**MIB:ZPL:00287**	2407	*Myotis myotis* (Borkhausen, 1797)	Vespertilionidae	**FR856695**	Onferno Natural Reserve (RN)	NIT
**MIB:ZPL:00300**	4407	*Myotis myotis* (Borkhausen, 1797)	Vespertilionidae	**FR856696**	Onferno Natural Reserve (RN)	NIT
**MIB:ZPL:00338**	5307	*Myotis myotis* (Borkhausen, 1797)	Vespertilionidae	**FR856697**	Onferno Natural Reserve (RN)	NIT
**MIB:ZPL:00377**	5207	*Myotis myotis* (Borkhausen, 1797)	Vespertilionidae	**FR856698**	Onferno Natural Reserve (RN)	NIT
**MIB:ZPL:01336**	3207	*Myotis myotis* (Borkhausen, 1797)	Vespertilionidae	**FR856704**	Onferno Natural Reserve (RN)	NIT
**MIB:ZPL:01339**	3107	*Myotis myotis* (Borkhausen, 1797)	Vespertilionidae	**FR856705**	Onferno Natural Reserve (RN)	NIT
**_**	_	*Myotis mystacinus* (Kuhl, 1817)	Vespertilionidae	**GU270555**	_	IE
**_**	_	*Myotis mystacinus* (Kuhl, 1817)	Vespertilionidae	**GU270556**	_	IE
**_**	_	*Myotis mystacinus* (Kuhl, 1817)	Vespertilionidae	**GU270557**	_	IE
**_**	_	*Myotis mystacinus* (Kuhl, 1817)	Vespertilionidae	**GU270558**	_	DE
**_**	_	*Myotis mystacinus* (Kuhl, 1817)	Vespertilionidae	**GU270559**	_	FR
**MIB:ZPL:01240**	MYMYS44B	*Myotis mystacinus* (Kuhl, 1817)	Vespertilionidae	**FR856707**	Villavallelonga (AQ)	CIT
**MIB:ZPL:01293**	MYMYS94B	*Myotis mystacinus* (Kuhl, 1817)	Vespertilionidae	**FR856708**	Cilento (SA)	SIT
**MIB:ZPL:02290**	209	*Myotis mystacinus* (Kuhl, 1817)	Vespertilionidae	**FR856710**	Coghinas (SS)	SAR
**MIB:ZPL:02289**	205	*Myotis mystacinus* (Kuhl, 1817)	Vespertilionidae	**FR856709**	Rio Calaresu (NU)	SAR
**MIB:ZPL:03775**	MYOMIS-0010	*Myotis mystacinus* (Kuhl, 1817)	Vespertilionidae	**FR856706**	Robecco sul Naviglio (MI)	NIT
_	_	*Myotis nattereri* (Kuhl, 1817)	Vespertilionidae	GU270561	_	UK
MIB:ZPL:01233	MYNA37B	*Myotis nattereri* (Kuhl, 1817)	Vespertilionidae	FR856718	Villavallelonga (AQ)	CIT
MIB:ZPL:00505	0208	*Myotis nattereri* (Kuhl, 1817)	Vespertilionidae	FR856717	Prevalle (BS)	NIT
MIB:ZPL:00356	9207	*Myotis nattereri* (Kuhl, 1817)	Vespertilionidae	FR856714	Parco del Campo dei Fiori (VA)	NIT
MIB:ZPL:00383	9207	*Myotis nattereri* (Kuhl, 1817)	Vespertilionidae	FR856715	Parco del Campo dei Fiori (VA)	NIT
MIB:ZPL:00318	1707	*Myotis nattereri* (Kuhl, 1817)	Vespertilionidae	FR856711	Cariadeghe (BS)	NIT
MIB:ZPL:00326	9007	*Myotis nattereri* (Kuhl, 1817)	Vespertilionidae	FR856712	Cariadeghe (BS)	NIT
MIB:ZPL:00331	2307	*Myotis nattereri* (Kuhl, 1817)	Vespertilionidae	FR856713	Cariadeghe (BS)	NIT
MIB:ZPL:00384	2107	*Myotis nattereri* (Kuhl, 1817)	Vespertilionidae	FR856716	Cariadeghe (BS)	NIT
MIB:ZPL:01347	2607	*Myotis nattereri* (Kuhl, 1817)	Vespertilionidae	FR856731	Cariadeghe (BS)	NIT
MIB:ZPL:01273	MYNA74B	*Myotis nattereri* (Kuhl, 1817)	Vespertilionidae	FR856721	Corleto Monforte (SA)	SIT
MIB:ZPL:01282	MYNA83B	*Myotis nattereri* (Kuhl, 1817)	Vespertilionidae	FR856722	Felitto (SA)	SIT
MIB:ZPL:01284	MYNA85B	*Myotis nattereri* (Kuhl, 1817)	Vespertilionidae	FR856723	Felitto (SA)	SIT
MIB:ZPL:01285	MYNA86B	*Myotis nattereri* (Kuhl, 1817)	Vespertilionidae	FR856724	Felitto (SA)	SIT
MIB:ZPL:01286	MYNA91B	*Myotis nattereri* (Kuhl, 1817)	Vespertilionidae	FR856725	Monte San Giacomo (SA)	SIT
MIB:ZPL:01291	MYNA92B	*Myotis nattereri* (Kuhl, 1817)	Vespertilionidae	FR856726	Monte San Giacomo (SA)	SIT
MIB:ZPL:01292	MYNA96B	*Myotis nattereri* (Kuhl, 1817)	Vespertilionidae	FR856727	Monte San Giacomo (SA)	SIT
MIB:ZPL:01308	MYMYS93B	*Myotis nattereri* (Kuhl, 1817)	Vespertilionidae	FR856728	Monte San Giacomo (SA)	SIT
MIB:ZPL:01309	MYNAS95B	*Myotis nattereri* (Kuhl, 1817)	Vespertilionidae	FR856729	Monte San Giacomo (SA)	SIT
MIB:ZPL:01249	MYNA53B	*Myotis nattereri* (Kuhl, 1817)	Vespertilionidae	FR856719	Cusano Mutri (CE)	SIT
MIB:ZPL:01266	MYNA67B	*Myotis nattereri* (Kuhl, 1817)	Vespertilionidae	FR856720	Castello del Matese (CE)	SIT
MIB:ZPL:01324	MYONA01B	*Myotis nattereri* (Kuhl, 1817)	Vespertilionidae	FR856730	Veio (Roma)	CIT

Voucher	Sample name	Species	Family	GenBank Accession Nos.	Locality	Locality group
**MIB:ZPL:01566**	207	*Myotis punicus* (Felten, 1977)	Vespertilionidae	**FR856732**	Sardinia	SAR
**MIB:ZPL:01567**	208	*Myotis punicus* (Felten, 1977)	Vespertilionidae	**FR856733**	Sardinia	SAR
**MIB:ZPL:01568**	209	*Myotis punicus* (Felten, 1977)	Vespertilionidae	**FR856734**	Sardinia	SAR
MIB:ZPL:01211	MYMYS15B	*Myotis* sp.	Vespertilionidae	FR856735	Villavallelonga (AQ)	CIT
MIB:ZPL:01214	MYMYS18B	*Myotis* sp.	Vespertilionidae	FR856736	Villavallelonga (AQ)	CIT
MIB:ZPL:01216	MYMYS20B	*Myotis* sp.	Vespertilionidae	FR856737	Villavallelonga (AQ)	CIT
MIB:ZPL:01221	MYMYS25B	*Myotis* sp.	Vespertilionidae	FR856738	Villavallelonga (AQ)	CIT
MIB:ZPL:01222	MYMYS26B	*Myotis* sp.	Vespertilionidae	FR856739	Villavallelonga (AQ)	CIT
MIB:ZPL:01223	MYMYS27B	*Myotis* sp.	Vespertilionidae	FR856740	Villavallelonga (AQ)	CIT
MIB:ZPL:01228	MYMYS32B	*Myotis* sp.	Vespertilionidae	FR856750	Villavallelonga (AQ)	CIT
MIB:ZPL:01230	MYMYS34B	*Myotis* sp.	Vespertilionidae	FR856751	Villavallelonga (AQ)	CIT
MIB:ZPL:01235	MYMYS39B	*Myotis* sp.	Vespertilionidae	FR856741	Villavallelonga (AQ)	CIT
MIB:ZPL:01281	MYMYS82	*Myotis* sp.	Vespertilionidae	FR856743	Corleto Monforte (SA)	SIT
MIB:ZPL:01287	MYMYS87B	*Myotis* sp.	Vespertilionidae	FR856744	Felitto (SA)	SIT
MIB:ZPL:01289	MYMYS89B	*Myotis* sp.	Vespertilionidae	FR856745	Felitto (SA)	SIT
MIB:ZPL:01301	MYMYS180B	*Myotis* sp.	Vespertilionidae	FR856746	Borgorose (RI)	CIT
MIB:ZPL:01302	MYMYS181B	*Myotis* sp.	Vespertilionidae	FR856747	Borgorose (RI)	CIT
MIB:ZPL:01303	MYMYS182B	*Myotis* sp.	Vespertilionidae	FR856748	Borgorose (RI)	CIT
MIB:ZPL:01256	MYMYS59B	*Myotis* sp.	Vespertilionidae	FR856742	Cusano Mutri (CE)	SIT
MIB:ZPL:01319	MYMYS07B	*Myotis* sp.	Vespertilionidae	FR856749	Cusano Mutri (CE)	SIT
**_**	_	*Nyctalus leisleri* (Kuhl, 1817)	Vespertilionidae	**GU270566**	_	FR
**MIB:ZPL:01207**	NYLE11B	*Nyctalus leisleri* (Kuhl, 1817)	Vespertilionidae	**FR856755**	Villavallelonga (AQ)	CIT
**MIB:ZPL:01268**	NYLE69B	*Nyctalus leisleri* (Kuhl, 1817)	Vespertilionidae	**FR856756**	Corleto Monforte (SA)	SIT
**MIB:ZPL:00261**	_	*Nyctalus leisleri* (Kuhl, 1817)	Vespertilionidae	**FR856752**	Malcantone (CH)	SW
**MIB:ZPL:00535**	_	*Nyctalus leisleri* (Kuhl, 1817)	Vespertilionidae	**FR856753**	Malcantone (CH)	SW
**MIB:ZPL:00536**	_	*Nyctalus leisleri* (Kuhl, 1817)	Vespertilionidae	**FR856754**	Malcantone (CH)	SW
**MIB:ZPL:01323**	UMNL01B	*Nyctalus leisleri* (Kuhl, 1817)	Vespertilionidae	**FR856757**	Umbria	CIT
**MIB:ZPL:02259**	0009	*Nyctalus leisleri* (Kuhl, 1817)	Vespertilionidae	**FR856758**	Val Bodengo (SO)	NIT
**MIB:ZPL:01476**	MZUF 21485	*Nyctalus noctula* (Schreber, 1774)	Vespertilionidae	**FR856759**	Cervia (RA)	NIT
**MIB:ZPL:00240**	_	*Pipistrellus kuhlii* (Kuhl, 1817)	Vespertilionidae	**FR856760**	Parco del Campo dei Fiori (VA)	NIT
**MIB:ZPL:00289**	_	*Pipistrellus kuhlii* (Kuhl, 1817)	Vespertilionidae	**FR856762**	Parco del Campo dei Fiori (VA)	NIT
**MIB:ZPL:00253**	_	*Pipistrellus kuhlii* (Kuhl, 1817)	Vespertilionidae	**FR856761**	Parco del Campo dei Fiori (VA)	NIT
**MIB:ZPL:01251**	PIKU54B	*Pipistrellus kuhlii* (Kuhl, 1817)	Vespertilionidae	**FR856763**	Cusano Mutri (CE)	SIT
**MIB:ZPL:01297**	PIKU152B	*Pipistrellus kuhlii* (Kuhl, 1817)	Vespertilionidae	**FR856764**	Monte Navegna Natural Reserve (RI)	CIT
**MIB:ZPL:01312**	UM PKU1B	*Pipistrellus kuhlii* (Kuhl, 1817)	Vespertilionidae	**FR856765**	Umbria	CIT
**MIB:ZPL:01322**	PIKU02B	*Pipistrellus kuhlii* (Kuhl, 1817)	Vespertilionidae	**FR856766**	Veio (Roma)	CIT
**MIB:ZPL:01180**	0008	*Pipistrellus nathusii* (Keyserling & Blasius, 1839)	Vespertilionidae	**FR856767**	Parco del Campo dei Fiori (VA)	NIT
**MIB:ZPL:01328**	0208	*Pipistrellus nathusii* (Keyserling & Blasius, 1839)	Vespertilionidae	**FR856768**	Parco del Campo dei Fiori (VA)	NIT
**MIB:ZPL:01331**	0108	*Pipistrellus nathusii* (Keyserling & Blasius, 1839)	Vespertilionidae	**FR856769**	Parco del Campo dei Fiori (VA)	NIT
**MIB:ZPL:01334**	0308	*Pipistrellus nathusii* (Keyserling & Blasius, 1839)	Vespertilionidae	**FR856770**	Parco del Campo dei Fiori (VA)	NIT
**MIB:ZPL:01224**	PIPIP28B	*Pipistrellus pipistrellus* (Schreber, 1774)	Vespertilionidae	**FR856776**	Villavallelonga (AQ)	CIT
**MIB:ZPL:01195**	0008	*Pipistrellus pipistrellus* (Schreber, 1774)	Vespertilionidae	**FR856775**	Campo Franscia (SO)	NIT
**MIB:ZPL:00272**	0507	*Pipistrellus pipistrellus* (Schreber, 1774)	Vespertilionidae	**FR856771**	Castelseprio (VA)	NIT

Voucher	Sample name	Species	Family	GenBank Accession Nos.	Locality	Locality group
**MIB:ZPL:00283**	0307	*Pipistrellus pipistrellus* (Schreber, 1774)	Vespertilionidae	**FR856772**	Castelseprio (VA)	NIT
**MIB:ZPL:00288**	0607	*Pipistrellus pipistrellus* (Schreber, 1774)	Vespertilionidae	**FR856773**	Castelseprio (VA)	NIT
**MIB:ZPL:00291**	1007	*Pipistrellus pipistrellus* (Schreber, 1774)	Vespertilionidae	**FR856774**	Castelseprio (VA)	NIT
**MIB:ZPL:01337**	0807	*Pipistrellus pipistrellus* (Schreber, 1774)	Vespertilionidae	**FR856779**	Castelseprio (VA)	NIT
**MIB:ZPL:01290**	PIPI90B	*Pipistrellus pipistrellus* (Schreber, 1774)	Vespertilionidae	**FR856777**	Felitto (SA)	SIT
**MIB:ZPL:02253**	0309	*Pipistrellus pipistrellus* (Schreber, 1774)	Vespertilionidae	**FR856780**	Gravedona (CO)	NIT
**MIB:ZPL:02254**	0409	*Pipistrellus pipistrellus* (Schreber, 1774)	Vespertilionidae	**FR856781**	Gravedona (CO)	NIT
**MIB:ZPL:02255**	0109	*Pipistrellus pipistrellus* (Schreber, 1774)	Vespertilionidae	**FR856782**	Gravedona (CO)	NIT
**MIB:ZPL:01321**	PIPIP08B	*Pipistrellus pipistrellus* (Schreber, 1774)	Vespertilionidae	**FR856778**	Cusano Mutri (CE)	SIT
**MIB:ZPL:02287**	194	*Pipistrellus pygmaeus* (Leach, 1825)	Vespertilionidae	**FR856784**	Seui (OG)	SAR
**MIB:ZPL:02285**	501	*Pipistrellus pygmaeus* (Leach, 1825)	Vespertilionidae	**FR856783**	Sorso (SS)	SAR
MIB:ZPL:01239	PPYG43B	*Pipistrellus* sp.	Vespertilionidae	FR856799	Villavallelonga (AQ)	CIT
MIB:ZPL:01241	PPYG45B	*Pipistrellus* sp.	Vespertilionidae	FR856800	Villavallelonga (AQ)	CIT
MIB:ZPL:02288	502	*Pipistrellus* sp.	Vespertilionidae	FR856801	Calagonone (NU)	SAR
MIB:ZPL:03815	p.pip-001	*Pipistrellus* sp.	Vespertilionidae	FR856785	Bosco della Fontana (MN)	NIT
MIB:ZPL:03816	p.pip-002	*Pipistrellus* sp.	Vespertilionidae	FR856786	Bosco della Fontana (MN)	NIT
MIB:ZPL:03817	p.pip-003	*Pipistrellus* sp.	Vespertilionidae	FR856787	Bosco della Fontana (MN)	NIT
MIB:ZPL:03818	p.pyg-001	*Pipistrellus* sp.	Vespertilionidae	FR856788	Bosco della Fontana (MN)	NIT
MIB:ZPL:03819	p.pyg-002	*Pipistrellus* sp.	Vespertilionidae	FR856789	Bosco della Fontana (MN)	NIT
MIB:ZPL:03820	p.pyg-003	*Pipistrellus* sp.	Vespertilionidae	FR856790	Bosco della Fontana (MN)	NIT
MIB:ZPL:03821	p.pyg-004	*Pipistrellus* sp.	Vespertilionidae	FR856791	Bosco della Fontana (MN)	NIT
MIB:ZPL:03822	p.pyg-005	*Pipistrellus* sp.	Vespertilionidae	FR856792	Bosco della Fontana (MN)	NIT
MIB:ZPL:03823	p.pyg-006	*Pipistrellus* sp.	Vespertilionidae	FR856793	Bosco della Fontana (MN)	NIT
MIB:ZPL:03824	p.pyg-007	*Pipistrellus* sp.	Vespertilionidae	FR856794	Bosco della Fontana (MN)	NIT
MIB:ZPL:03825	p.pyg-008	*Pipistrellus* sp.	Vespertilionidae	FR856795	Bosco della Fontana (MN)	NIT
MIB:ZPL:03826	p.pyg-009	*Pipistrellus* sp.	Vespertilionidae	FR856796	Bosco della Fontana (MN)	NIT
MIB:ZPL:03827	p.pyg-010	*Pipistrellus* sp.	Vespertilionidae	FR856797	Bosco della Fontana (MN)	NIT
MIB:ZPL:03828	p.pyg-011	*Pipistrellus* sp.	Vespertilionidae	FR856798	Bosco della Fontana (MN)	NIT
**MIB:ZPL:01206**	PLAUR10B	*Plecotus auritus* (Linnaeus, 1758)	Vespertilionidae	**FR856804**	Villavallelonga (AQ)	CIT
**MIB:ZPL:01227**	PLAUR31B	*Plecotus auritus* (Linnaeus, 1758)	Vespertilionidae	**FR856805**	Villavallelonga (AQ)	CIT
**MIB:ZPL:01345**	0307	*Plecotus auritus* (Linnaeus, 1758)	Vespertilionidae	**FR856809**	Parco del Campo dei Fiori (VA)	NIT
**MIB:ZPL:01346**	0207	*Plecotus auritus* (Linnaeus, 1758)	Vespertilionidae	**FR856810**	Parco del Campo dei Fiori (VA)	NIT
**MIB:ZPL:01190**	0108	*Plecotus auritus* (Linnaeus, 1758)	Vespertilionidae	**FR856803**	Cariadeghe (BS)	NIT
**MIB:ZPL:01269**	PLAUR70B	*Plecotus auritus* (Linnaeus, 1758)	Vespertilionidae	**FR856807**	Corleto Monforte (SA)	SIT
**MIB:ZPL:01276**	PLAUR77B	*Plecotus auritus* (Linnaeus, 1758)	Vespertilionidae	**FR856808**	Corleto Monforte (SA)	SIT
**MIB:ZPL:01253**	PLAUR56B	*Plecotus auritus* (Linnaeus, 1758)	Vespertilionidae	**FR856806**	Cusano Mutri (CE)	SIT
**MIB:ZPL:00378**	0107	*Plecotus auritus* (Linnaeus, 1758)	Vespertilionidae	**FR856802**	San Martino (LC)	NIT
**MIB:ZPL:01497**	MZUF 21381	*Plecotus austriacus* (Fischer, 1829)	Vespertilionidae	**FR856812**	Firenze (FI)	CIT
**MIB:ZPL:01252**	PLAUS55B	*Plecotus austriacus* (Fischer, 1829)	Vespertilionidae	**FR856811**	Cusano Mutri (CE)	SIT
**MIB:ZPL:00256**	_	*Plecotus macrobullaris* (Kusjakin, 1965)	Vespertilionidae	**FR856813**	Trento (TN)	NIT
**MIB:ZPL:01200**	0108	*Plecotus macrobullaris* (Kusjakin, 1965)	Vespertilionidae	**FR856814**	Campo Moro (SO)	NIT
**MIB:ZPL:01572**	213	*Plecotus sardus* Mucedda, Kiefer, Pidinchedda, Veith, 2002	Vespertilionidae	**FR856815**	Sardinia	SAR
**MIB:ZPL:01574**	215	*Plecotus sardus* Mucedda, Kiefer, Pidinchedda, Veith, 2002	Vespertilionidae	**FR856816**	Sardinia	SAR

**Voucher**	**Sample name**	**Species**	**Family**	**GenBank Accession Nos.**	**Locality**	**Locality group**
**MIB:ZPL:01575**	216	*Plecotus sardus* Mucedda, Kiefer, Pidinchedda, Veith, 2002	Vespertilionidae	**FR856817**	Sardinia	SAR
MIB:ZPL:03414	_	*Plecotus* sp.	Vespertilionidae	FR856824	Santorso (VI)	NIT
MIB:ZPL:00262	_	*Plecotus* sp.	Vespertilionidae	FR856818	Trento (TN)	NIT
MIB:ZPL:01189	0008	*Plecotus* sp.	Vespertilionidae	FR856823	Campo Moro (SO)	NIT
MIB:ZPL:00265	0106	*Plecotus* sp.	Vespertilionidae	FR856819	Monte Pravello (VA)	NIT
MIB:ZPL:00268	0006	*Plecotus* sp.	Vespertilionidae	FR856820	Monte Pravello (VA)	NIT
MIB:ZPL:00269	0107	*Plecotus* sp.	Vespertilionidae	FR856821	Monte San Martino (LC)	NIT
MIB:ZPL:00270	0007	*Plecotus* sp.	Vespertilionidae	FR856822	Monte San Martino (LC)	NIT
