# Supplementary material for: Integrated Operational Taxonomic Units (IOTUs) in Echolocating Bats: A Bridge between Molecular and Traditional Taxonomy
Source: PLoS One. 2012 Jun 28;7(6):e40122. doi: 10.1371/journal.pone.0040122 (PMC3386196; doi:10.1371/journal.pone.0040122)
Supplement: Table S2 — List of GenBank accession numbers and sampling details of bats belonging to the M. nattereri species complex. Sampling locality details, accession numbers with related reference of cyt b and ND1 sequences and name of corresponding lineage are provided for each individual. (PDF) [file pone.0040122.s006.pdf]

		GenBank Accession Nos.			
**Species / Voucher**	**Sampling Locality and abbreviation**	***Cyt b***	***ND1***	**Lineage**	**Reference**
Myotis nattereri MIB:ZPL:01249	Southern Italy (SIT)	_	FR856852	*Myotis* sp. C	this study
Myotis nattereri MIB:ZPL:01282	Southern Italy (SIT)	_	FR856853	*Myotis* sp. C	this study
Myotis nattereri MIB:ZPL:01324	Southern Italy (SIT)	FR856847	FR856854	*Myotis* sp. C	this study
Myotis nattereri sp. B	Southern Morocco (SMO)	EU360644	EU360612	*Myotis* sp. B	[25]
Myotis nattereri sp. B	Central Morocco (CMO)	EU360645	_	*Myotis* sp. B	[25]
Myotis nattereri sp. B	Central Morocco (CMO)	EU360646	_	*Myotis* sp. B	[25]
Myotis nattereri sp. B	Northern Morocco/Central Morocco (NMO CMO)	EU360647	_	*Myotis* sp. B	[25]
Myotis nattereri sp. B	Northern Morocco (NMO)	EU360648	EU360613	*Myotis* sp. B	[25]
Myotis nattereri sp. A	Northern Iberia (NIB)	DQ120884	_	*Myotis* sp. A	[57]
Myotis nattereri sp. A	Northern Iberia (NIB)	DQ120886	DQ120801	*Myotis* sp. A	[57]
Myotis nattereri sp. A	Northern Iberia (NIB)	DQ120885	_	*Myotis* sp. A	[57]
Myotis nattereri sp. A	Austria (AUS)	_	DQ915049	*Myotis* sp. A	[32]
Myotis nattereri MIB:ZPL:00331	Northern Italy (NIT)	_	FR856851	*Myotis* sp. A	this study
Myotis nattereri MIB:ZPL:00356	Northern Italy (NIT)	_	FR856849	*Myotis* sp. A	this study
Myotis nattereri MIB:ZPL:01347	Northern Italy (NIT)	FR856848	FR856850	*Myotis* sp. A	this study
Myotis nattereri	Switzerland (SWI)	DQ120892	_	*Myotis nattereri*	[57]
Myotis nattereri	Germany (GER)	DQ120893	_	*Myotis nattereri*	[57]
Myotis nattereri	Germany (GER)	DQ120894	_	*Myotis nattereri*	[57]
Myotis nattereri	Germany (GER)	DQ120895	_	*Myotis nattereri*	[57]
Myotis nattereri	Greece (GRE)	AF376863	AY033984	*Myotis nattereri*	[61]
Myotis nattereri	Hungary (HUN)	_	AF401439	*Myotis nattereri*	[59]
Myotis escalerai	Southern Iberia (SIB)	DQ120890	EU360614	*Myotis escalerai*	[25]
Myotis escalerai	Southern Iberia (SIB)	DQ120890	EU360615	*Myotis escalerai*	[25]
Myotis escalerai	Southern Iberia (SIB)	DQ120891	DQ120802	*Myotis escalerai*	[25]
Myotis escalerai	Southern Iberia (SIB)	EU360649	_	*Myotis escalerai*	[25]
Myotis escalerai	Northern Iberia (NIB)	DQ120887	_	*Myotis escalerai*	[57]
Myotis escalerai	Northern Iberia (NIB)	DQ120888	_	*Myotis escalerai*	[57]
Myotis Myotis	Northern Iberia (NIB)	AF246241	DQ120800	_	[37]
